# Correction: Laser-Supported CD133+ Cell Therapy in Patients with Ischemic Cardiomyopathy: Initial Results from a Prospective Phase I Multicenter Trial

**DOI:** 10.1371/journal.pone.0107082

**Published:** 2014-08-25

**Authors:** The PLOS ONE Staff

## Notice of Republication

This article was republished on August 7, 2014, to replace incorrectly changed characters in the byline. The publisher apologizes for the error. Please download this article again to view the correct version. The originally published, uncorrected article and the republished, corrected article are provided here for reference.

## Supporting Information

File S1 Originally published, uncorrected article.
(PDF)

File S2 Republished, corrected article.
(PDF)
