# Supplementary material for: Laser-Supported CD133+ Cell Therapy in Patients with Ischemic Cardiomyopathy: Initial Results from a Prospective Phase I Multicenter Trial
Source: PLoS One. 2014 Jul 7;9(7):e101449. doi: 10.1371/journal.pone.0101449 (PMC4084817; doi:10.1371/journal.pone.0101449)
Supplement: Protocol S1 — Clinical trial protocol of this study. (PDF) [file pone.0101449.s001.pdf]

Department of Thoracic and Cardiovascular Surgery
Heinrich-Heine-University hospital Duesseldorf

Clinical Trial Protocol

Intramyocardial Application of Stem Cells in Combination with Transmyocardial Laser Revascularisation (TMLR) in CABG Patients (INSTEM-Trial)

3/2007

Coordinating Investigator: Prof. H.M. Klein MD
Department of Thoracic and Cardiovascular Surgery
Heinrich-Heine-Universityhospital Duesseldorf
Moorenstr. 5
40225 Duesseldorf,
Germany
Tel.: 0049 211 8118331
Fax.: 0049 211 8118333

Protocol-No.: Instem_HHU_2005

EudraCT-No.: 2005-004051-35

Version: V04_F01 3/2007

All information in this study protocol is strictly confidential. It is only to be used by the investigators, other collaborators of the study, the supporting company, the ethic committees, the authorities, and the patients. This study protocol shall not be distributed to third parties without written consent from the principle coordinating investigator.

© Klein / Coordination Centre for Clinical Trials Duesseldorf

C O N F I D E N T I A L

Front page

Title:	Intramyocardial Application of Stem Cells in Combination with Transmyocardial Laser Revascularisation (TMLR) in CABG Patients (INSTEM)
Indication:	Patients with coronary artery disease and the indication for a coronary artery bypass surgery (CABG)
Sponsor	Heinrich-Heine-University Duesseldorf<br>Universitätstr. 1,<br>40225 Duesseldorf, Germany<br>represented by the Coordinating Investigator
Principal/ Coordinating Investigator	Prof. H.M. Klein MD<br>Department of Thoracic and Cardiovascular Surgery<br>Heinrich-Heine-Universityhospital Duesseldorf<br>Moorenstr. 5<br>40225 Duesseldorf, Germany<br>Tel.: 0049 211 8118331<br>Fax.: 0049 211 8118333<br>kleinmi@uni-duesseldorf.de
Financial Advancement	1.) PLC Sistemas medicos Internacionais (Germany) GmbH<br>Hochallee 11<br>20149 Hamburg<br>Germany<br>2.) Miltenyi Biotec GmbH<br>Friedrich-Ebert-Str. 68<br>51429 Bergisch Gladbach<br>Germany
Protocoll-No.	Instem_HHU_2005
EUDRACT No.	2005-004051-35
Phase	II
Time Schedule	First patient first Visit 01.04.2007
	Last patient in: 01.04.2008
	Last patient last visit: 01.04.2009
	Database close: 01.06.2009
	Final statistical analysis: 01.09.2009
Co-Investigator:	Prof. Axel Haverich MD<br>Prof. M. Karck MD<br>A. Ruhparwar MD<br>Department of Cardiac and Thoracic Surgery<br>Medical School of Hanover<br>Carl Neuberg-Str.1<br>30625 Hannover<br>Tel.: 0049-511-5320<br>Fax: 49-(0)511-532 54 04

© Klein / Coordination Centre for Clinical Trials Duesseldorf

C O N F I D E N T I A L

Co-Investigator: (continued)	PD Dr. K.F. Wagner MD<br>Prof. H.H. Sievers, MD<br>Clinics of Anaesthesiology and Cardiac Surgery<br>Universitätsklinikum Schleswig-Holstein<br>Ratzeburger Allee 160<br>D-23538 Lübeck<br>Tel.: 0049-451-500-4057<br>Fax.: 0049-451-500-3405
Protocol development	Prof.H.M. Klein MD<br>Prof.Ch. Ohmann, PhD<br>H. Kolbe<br>S. Sell
Statistics	Prof. Ch. Ohmann, PhD<br>P. Verde, PhD
Ethics committee	Ethics committee<br>Heinrich Heine-University Duesseldorf (principal)<br>Ethics committee Lübeck (local)<br>Ethics committee Hannover (local)
Study Unit	Coordination Centre for Clinical Trials<br>Heinrich Heine University Duesseldorf<br>Moorenstr. 5, 40225 Duesseldorf

© Klein / Coordination Centre for Clinical Trials Duesseldorf

C O N F I D E N T I A L

1 Signatures 8

2 Co-Investigator Signatures 9

3 Synopsis 10

4 Flowchart 13

5 Introduction/Background 14

6 Aim of Study 14

7 Endpoints 15

7.1 Primary Endpoint 15

7.2 Secondary Endpoints 16

8 Subject Selection 17

8.1 Design of the Study 17

8.2 Inclusion criteria 17

8.3 Exclusion criteria 17

8.4 Drop out criteria 18

9 Registration 18

10 Treatment 19

11 Stem cell harvest and cell preparation 19

11.1 Introduction/Background 19

11.2 CD133+ stem cell preparation 19

11.3 CliniMACS Technology 20

12 Investigational Procedure 21

12.1 CABG surgery 21

13 Assessment of Efficacy / Safety Data 25

13.1 Overview 25

13.2 Screening of patients 25

13.3 Pre-operative Assessments 26

© Klein / Coordination Centre for Clinical Trials Duesseldorf

13.4 Operative procedure 26

14 Patient Follow-Up - Post-Operative & Pre-Discharge 27

15 Medical Treatment of the patients after completion of the trial 28

16 Adverse Events 30

16.1 Adverse Event Monitoring 30

16.2 Adverse Event Definitions 30

16.3 Relationship of Adverse Event to Investigational Procedure 32

16.4 Documentation of Adverse Events 32

16.5 Reporting of Serious Adverse Events 33

16.6 Follow-up of patient sustaining AEs/SAEs 33

16.7 Notification of the Ethics Committee and Competent Authority 34

16.8 Information of the Investigator 34

16.9 Annual safety reports 35

17 Duration of Study / End of Study 35

18 Statistics 35

18.1 Sample Size 35

18.2 Study sites 36

18.3 Statistical Analysis 36

19 Data Management 36

19.1 Administrative Aspects of the Investigational Study 36

19.2 Data Control Methods 36

19.3 Data Management 37

20 Clinical Monitoring 37

21 Record Retention 37

22 Ethics 38

23 Ethics Committee/Institutional Review Board 38

© Klein / Coordination Centre for Clinical Trials Duesseldorf

C O N F I D E N T I A L

24 Protocol Modification 38

25 Patient Informed Consent 39

26 Insurance 39

27 Publication Policy 39

28 List of Abbreviations 40

29 References 41

Supplements 42

MACE (Major adverse cardiac event) 42
MI definition 42
Canadian Cardiovascular Society Grading Scale for Angina Pectoris 43
Laboratory parameters 44
CTC-criteria for cardiac AEs 45
LASER Treatment 48

Appendix

All dokuments exist in separate form, they are still official part of this study protocol.

Appendix A: INSTEM Study table (study protocol based handout)

Appendix B: Fachinformation ratiopharm Heparin und ratiopharm ASS

Appendix C: SAE-Form

Appendix D: informed consent

Appendix E: Insurance

Appendix F: Seattle Angina Questionaire

© Klein / Coordination Centre for Clinical Trials Duesseldorf

C O N F I D E N T I A L

1 Signatures

**Sponsor**<br>Heinrich-Heine-University, Duesseldorf<br>represented by the Coordinating Investigator	Signature	Date
**Prof. Dr. Klein MD**<br>Coordinating Investigator<br>Department of Thoracic and Cardiovascular Surgery<br>Heinrich-Heine University Duesseldorf	Signature	Date
**Prof. Dr. Gams**<br>Head of Department<br>Department of Thoracic and Cardiovascular Surgery<br>Heinrich-Heine University Duesseldorf	Signature	Date
**Prof. Dr. Ohmann**<br>Statistics<br>Coordination Center for Clinical trials (KKS)<br>Heinrich-Heine University Duesseldorf	Signature	Date

© Klein / Coordination Centre for Clinical Trials Duesseldorf

C O N F I D E N T I A L

2 Co-Investigator Signatures

I confirm to have read the present clinical trial protocol and to conduct the trial according to protocol and potential amendments, applicable regulatory requirements, Good Clinical Practice and the Declaration of Helsinki, 1996, Somerset West.

In addition, I will allow direct access to source documents and agree to inspection by auditors from the sponsor and regulatory authorities.

Name<br>(in block letters)	Signature	Date
Name<br>(in block letters)	Signature	Date
Name<br>(in block letters)	Signature	Date
Name<br>(in block letters)	Signature	Date

© Klein / Coordination Centre for Clinical Trials Duesseldorf

C O N F I D E N T I A L

3 Synopsis

Study Title	Intramyocardial Application of Stem Cells in Combination with Transmyocardial Laser Revascularisation (TMLR) in CABG Patients (INSTEM)
Aim of Study	A prospective study to assess safety and efficacy of stem cell application with regard to regional myocardial improvement in patients with CABG and TMLR
Acronym	INSTEM
Study Design	Prospective uncontrolled multicentre trial (Phase II)
Study Duration	12 months
Study end	01.10.2008
Center Numbers	3
Investigational Sites	Department of Thoracic and Cardiovascular Surgery<br>Heinrich-Heine-Universityhospital Duesseldorf<br>Moorenstr. 5<br>40225 Duesseldorf<br><br>Clinics of Anaesthesiology and Cardiac Surgery<br>Universitätsklinikum Schleswig-Holstein<br>Ratzeburger Allee 160<br>D-23538 Lübeck<br><br>Department of Cardiac and Thoracic Surgery<br>Medical School of Hanover<br>Carl Neuberg-str.1<br>D-30625 Hannover
Investigator	Duesseldorf<br>Prof. H. M. Klein MD<br>Hannover<br>Prof. A. Haverich MD<br>Prof. M. Karck MD<br>A. Ruhparwar MD<br>Lübeck<br>Dr. K. F. Wagner MD<br>Prof. H. H. Sievers MD
Number of patients	40
Inclusion criteria	• ≥ 18 years (male or female gender)<br>• Presence of at least two vessel coronary artery disease with at least one vessel that is not amenable to CABG, according to the angiogram, this vessel must serve an area of viable myocardium<br>• Area of interest defined as part of free left ventricular wall with reduced contractility as shown either in ventriculographia during angiography and/or preoperative echo<br>• Demonstration of reduced perfusion in the area of interest by cardiac MRI or CT<br>• Global ejection fraction ≥ 15 % and < 35%<br>• signed informed consent

Exclusion criteria

- Any condition that in the believe of the treating physician prevents successful stem cell collection or application (e.g. systemic infection, puncture for stem cell collection impossible)
- Any condition that may adversely affect bone marrow (such as malignancy or prior irradiation to the pelvic bone)
- Mitral valve insufficiency > II
- History of ventricular arrhythmia, not controlled by medication and/or AICD (Automatic Implantable Cardioverter Defibrillator) required
- Need of additional heart surgery (i.e. valve replacement)
- Emergency or salvage operation defined as within 48 hours of diagnosis
- Evidence of left ventricular thrombus
- Previous heart surgery within the last 6 months (excluding implantation of pace maker)
- History of symptomatic carotid disease (e.g. any TIA, PRIND, stroke) within the last 3 months prior to study intervention
- Increased CK (>3 times normal) in patients with unstable angina
- End stage renal disease (ESRD) defined as serum creatinine level > 3.5 mg/dL, or dialysis (renal replacement therapy)
- Concurrent active chemotherapy for cancer
- Life expectancy ≤ 2 years
- Platelet count < 100000/ µl
- Pregnancy
- Participation in other clinical trials in the last 30 days
- Active Hepatitis-infection
- HIV-infection
- Anemia
- Haemorrhagic Diathesis in medical history
- Sensitivity and incompatibility against used drugs or excipients (see Appendix B for Heparin, Aspirin)
- Disseminated intravascular coagulation in medical history
- Clinically active infection at the time of operation
- Patient not able to attend follow-up as specified in the protocol
- No informed consent

Drop out criteria

Withdrawal of consent

Time Schedule

First patient first Visit	01.04.2007
Last patient in:	01.04.2008
Last patient last visit:	01.04.2009
Database close:	01.06.2009
Final statistical analysis:	01.09.2009

© Klein / Coordination Centre for Clinical Trials Duesseldorf

C O N F I D E N T I A L

Endpoints	Primary Endpoint<br>• Safety<br>Occurrence of MACE (Major Adverse Cardiac Event) assessed at three months after surgical study treatment. MACE will be assessed by the investigator for relationship to the interventions under the investigation in this trial.<br>Secondary Endpoints (assessed at 3, 6 and 12 months follow-up)<br>• MACE (Major Adverse Cardiac Event) assessed at 6 and 12 months follow-up<br>• Cardiac AEs defined in the Common Toxicity Criteria (CTC) (assessed form onset of surgery up to 12 months follow-up)<br>• Severity of angina and extent of treatment in comparison to baseline (CCS Classification) (assessed at 3, 6 and 12 months follow-up)<br>• Quality of Life in comparison to baseline (increase of exercise tolerance in Seattle Angina Questionnaire) (assessed at 3, 6 and 12 months follow-up)<br>• Baseline Regional cardiac function by Cardiac MRI is assessed and in comparison to Cardiac MRI at 6 months follow-up (In case of an contraindication against cardiac MRI the cardiac function is assessed by cardiac CT)
Therapy	• Patients will receive CABG (coronary artery bypass graft) with TMLR and autologous stem cell transplantation
Parameters and Methods	• Regional cardiac contractility: assessed by cardiac MRI or CT<br>• Severity and location of coronary artery disease assessed by coronary angiography<br>• Cardiac advers events defined in the Common Toxicity Criteria<br>• Free ventricular wall with reduced regional contractility assessed by echocardiography or/and ventriculography<br>• MACE: as defined in the Supplement<br>• Hybernating myocardial infarction assessed by cardiac MRI or CT<br>• Global ejection fraction assessed by echocardiography<br>• Severity of mitral valve insufficiency assessed by echocardiography or/and ventriculography according to PISA (proximal isovelocity surface area)- classification<br>• Severity of angina assessed by the modified Canadian Cardiovascular Society Grading Scale for Angina Pectoris (CCS, defined in the Supplement)<br>• Quality of Life (increase of exercise tolerance in Seattle Angina Questionnaire, defined in Appendix F)

© Klein / Coordination Centre for Clinical Trials Duesseldorf

CONFIDENTIAL

4 Flowchart

Clinical examination and surgical strategy

↓

Day - 1 Preoperativ assessment whether transplantation of stem cells and TMLR is appropriate; cardiac MRI /or CT and echocardiography (within one month before surgery)

↓

Day 0 Stem cell collection and processing

↓

CABG, TMLR
and
stem cell transplantation

↓

Day 4-7 Postoperative evaluation/ pre-discharge assessment

↓

3 months Evaluation with regard to primary and secondary endpoints

↓

6 months Evaluation with regard to secondary endpoints (including MRI or CT)

↓

12 months Evaluation with regard to secondary endpoints

© Klein / Coordination Centre for Clinical Trials Duesseldorf

C O N F I D E N T I A L

5 Introduction/Background

Although the surgical and interventional revascularization of the ischemic myocardium can treat angina, reduce risk of myocardial infarction, and improve function of viable myocardium, viability of necrotic myocardium cannot be restored. Despite major advances in the treatment of ischemic heart disease, complete revascularisation procedures do not help a large number of patients because of poor distal vessels, total arterial occlusion, or unacceptable procedural risks due to concomitant medical conditions. Up to 15% of the patients with end-stage coronary artery disease suffer from disabling anginal symptoms regardless of maximal pharmacotherapy and conventional revascularisations (16). Cardiac remodeling has been implicated in the progression of heart disease. Although prompt reperfusion after acute myocardial infarction has reduced mortality, in patients who survive, heart muscle cells enlarge to compensate for the loss in heart pump function. If there is no increase in oxygen and nutrients these cells will die, and heart muscle will be lost. To restore tissue viability in ischemic myocardium not amenable to coronary bypass grafting transmyocardial LASER revascularization (TMLR) and recently transplantation of bone marrow derived stem cell (BMC) have been used in clinical setting(1,3,10,13,14).

TMLR was currently approved by FDA for patients with disabling angina, whose coronary arteries are not amenable to angioplasty or coronary artery bypass grafting alone and for patients with microvascular disease (1,3,16). Recent clinical and experimental trials have shown that symptomatic improvement after TMLR in patients with refractory angina is probably related to neoangiogenesis (5).

Implantation of bone-marrow stem cells into the heart is reported to be a feasible method for myocardial regeneration after myocardial infarction (2).

The aim of cell transplantation is to repopulate diseased myocardium with cells that could restore contractility. Results of experimental studies (4,6,9,10) have shown that bone marrow derived stem cells can be used to regenerate cardiomyocytes and induce angiogenesis after myocardial infarction, resulting in improvement of myocardial function. Results from animal models have shown that cardiomyocytes, myoblasts, and smooth-muscle cells can survive in, and improve contractility of ischemic myocardium (2,7,8,18).

CD 133+ is a recently discovered marker for more primitive multipotent stem and endothelial progenitor cells and of particular interest in studies directed to therapeutic angiogenesis, as these cells have been shown to differentiate into endothelial and myogenic cell lines (4).

Patients who received CD 133+ cells showed improved perfusion at injection sites of stem cells leading to a significant increase in volume of left ventricular ejection fraction, regional wall motion in the infarct zone, and a reduction in end systolic left ventricular volume (1,5).

The transplantation of MNCs (mononuclear cells) was reported to improve cardiac function (6), but when transplanting the whole MNC population, a much higher number of cells must be injected to achieve comparable effect as only less than 1,5% of CD 133+ is to be expected among the MNCs (6).

6 Aim of Study

The objective of this study is to assess safety and efficacy of stem cell application with regard to regional myocardial improvement in patients with CABG and TMLR.

© Klein / Coordination Centre for Clinical Trials Duesseldorf

C O N F I D E N T I A L

7 Endpoints

7.1 Primary Endpoint:

7.1.1. Safety

Safety is defined as the occurrence of MACE (Major adverse cardiac event) within three months after surgical study treatment.
For this study Major adverse cardiac events (MACE) are defined as :

- Death
- Myocardial infarction (MI) (definition below)
- Traumatic Mitral valve damage
- Urgent or elective PCI (percutaneous coronary intervention) and repeated coronar surgery
- Stroke/TIA
- Any hospitalisation, according to cardiac symptoms

MACE will be assessed by the investigator for relationship to the interventions under investigation in this trial.

MI definition:

**Timepoint**	**CK-MB criteria**	**ECG** (definition below)
Predischarge after bypass surgery	Not according to lab	Q-wave
associated with PCI	> 3 ULN	Q-wave, non-Q-wave
Spontaneous MI	> 2 ULN	Q-wave, non-Q-wave

ECG changes of Q-wave MI:
Any QR wave in leads V1 through V3 ≥ 30 ms; abnormal Q wave in lead I, II, aVL, aVF or V4 through V6 in any two contiguous leads and at least 1 mm in depth.

In the absence of QRS confounders (e.g. bundle branch block, left ventricular hypertrophy, Wolff-Parkinson-White syndrome)

ECG changes of Non-Q wave
In the presence of a new CK/CK-MB increase, a ST segment elevation ≥ 1 mm, ST segment depression ≥ 1 mm, or T wave inversion ≥ 1 mm in at least two contiguous leads (i.e. II, III, avF; I, aVL, V5, V6, or V1-V5)

© Klein / Coordination Centre for Clinical Trials Duesseldorf

CONFIDENTIAL

7.2 Secondary Endpoints

In this study the secondary endpoints are defined as :

7.2.1 MACE (Major adverse cardiac event)

Major adverse cardiac event assessed at 6 and 12 months follow-up as defined chapter 7.1 (Primary endpoint).

7.2.2 Cardiac Adverse Events

Clinical assessment of all cardiac AEs including cardiac SAEs. A complete safety analysis will be performed that encompasses all AEs (including SAEs) for onset of the study up to 12 months follow-up. All AEs, which are defined in the Common Toxicity Criteria (see Supplement) are considered and analysed.

7.2.3 Quality of Life (Seattle Angina Questionnaire)

Measurement of “Quality of life” as a disease-specific self-administered functional status measure for patients.
The Seattle Angina Questionaire (see Appendix F) is a frequently used instrument in cardiovascular research and widely accepted. The instrument is comprised of 11 questions that quantify clinically relevant domains of coronary artery disease. Analysis constitutes a relevant patient centred multifactor endpoint. The Questionaire will be accomplished by physician at screening/enrollment of the patient and again at 3, 6 and 12 months follow-up.

7.2.1 Reduction of classes in the Canadian Cardiovascular Society Grading Scale

Reduction of classes in the Canadian Cardiovascular Society Grading Scale

The Canadian Cardiovascular Society Grading Scale is a clinically relevant physician centred measure of the patient’s well-being. It is highly observer dependent, but generally accepted and introduced as well as absolutely non interventional.

7.2.2 Comparison according to extent of treatment and severity of angina

Comparison according to extent of treatment and severity of angina

This comparison will be used in an exploratory fashion to establish the group of patients that has the most benefit from the procedure. This will be done by exploratory subgroup analysis for severity of angina and the size of the treatment area.

© Klein / Coordination Centre for Clinical Trials Duesseldorf

C O N F I D E N T I A L

7.2.3 Regional cardiac function

Improvement of regional cardiac function is assessed by cardiac MRI (assessed only at 6 months). In case of an contraindication against cardiac MRI the endpoint is assessed by cardiac CT.

8 Subject Selection

The patient selection is responsibility of the treating surgeon, who will review the data and decide about eligibility of the patient; this is especially relevant in cases of divergent results from previous investigations. However, it is necessary that eligibility criteria can be confirmed by source data for all patients.

8.1 Design of the Study

The design of this study is based on available evidence. So far only data from a case serious of five patients are available investigating the combination of CABG, TMLR and stem cell application comparable to our trial. As a next step this uncontrolled multicentre study (Phase II) has been designed in order to evaluate safety and efficacy in a serious of 40 patients. Data will be used to evaluate outcome criteria which may be relevant for a later controlled clinical trial.

8.2 Inclusion criteria

- ≥ 18 years (male or female gender)
- Presence of at least two vessel coronary artery disease, with at least one vessel that is not amenable to CABG, according to the angiogram. This vessel must serve an area of viable myocardium
- Area of interest defined as part of free left ventricular wall with reduced contractility as shown either in ventriculographia during angiography and/or preoperative echo
- Demonstration of reduced perfusion in the in the area of interest by cardiac MRI or CT
- Global ejection fraction ≥ 15 % and < 35%
- signed informed consent

8.3 Exclusion criteria

- Any condition that in the believe of the treating physician prevents successful stem cell collection or application (e.g. systemic infection, puncture for stem cell collection impossible)
- Any condition that may adversely affect bone marrow (such as malignancy or prior irradiation to the pelvic bone)
- mitral valve insufficiency > II
- history of ventricular arrhythmia, not controlled by medication and/or AICD (Automatic Implantable Cardioverter Defibrillator) required
- need of additional heart surgery (i.e. valve replacement)

© Klein / Coordination Centre for Clinical Trials Duesseldorf

C O N F I D E N T I A L

- emergency or salvage operation defined as within 48 hours of diagnosis
- evidence of left ventricular thrombus
- previous heart surgery within 6 months (excluding implantation of pace maker)
- history of symptomatic carotid disease (e.g. any TIA, PRIND, stroke) within the last 3 months prior to study intervention
- increased CK (>3 times normal) in patients with unstable angina
- end stage renal disease (ESRD) defined as serum creatinine level > 3.5 mg/dL, or dialysis (renal replacement therapy)
- concurrent active chemotherapy for cancer
- life expectancy ≤ 2 years
- platelet count < 100000/ µl
- Pregnancy
- Participation in other clinical trails in the last 30 days
- active Hepatitis infection
- HIV-infection
- Anemia
- Haemorrhagic diathesis in medical history
- Sensitivity and incompatibility against used drugs or excipient (see appendix B for Heparin, Aspirin)
- Disseminated intravascular coagulation in medical history
- clinically active infection at the time of operation
- not able to attend follow-up as specified in the protocol
- no informed consent

8.4 Drop out criteria

- withdrawal of informed consent

9 Registration

Patients are going to be registered at the treating centre where identification lists are kept and archived. They are to be registered pseudonymously by Fax form at the Coordination Centre for Clinical Trials (KKS) within 24h after enrolled in the study. The KKS is going to monitor recruitment as well as follow up

Coordination Centre for Clinical Trials
Heinrich-Heine-University Hospital
Moorenstr. 5
40225 Duesseldorf
Fax + 49 211-81-19705

10 Treatment

A pre-study visit by the principal investigator at the participating sites will assure that the quality of surgical procedure is up to the standard necessary to safeguard the patients safety.

After having given informed consent the patient will be prepared for CABG surgery and bone marrow aspiration.
Care must be taken that definite preoperative eligibility is assessed before bone marrow aspiration is undertaken. Eligibility will be based on cardiologic parameters and the defined inclusion and exclusion criteria, the status of the coronary arteries, the perfusion and motility of different heart regions.

11 Stem cell harvest and cell preparation

11.1 Introduction/Background

CD133 is a recently discovered marker for primitive multipotent stem and endothelial progenitor cells angioblasts from peripheral blood, bone marrow, fetal liver and umbilical cord blood. CD133+ stem cells are of particular interest in studies investigating therapeutic angiogenesis, since these cells have been shown to differentiate into in-vitro and in-vivo endothelial cells. CD133+ in bone marrow are a rare and homogenous cell population with varying proliferative and developmental capability. Data from several laboratory support morphologic features of CD133-derived EPC consistent with vascular endothelial cultures. After short-term culture in media developed to expand vascular endothelial cells, many of these cells exhibit surface markers considered specific to endothelial cells including CD31 and P1H12. In culture, these cells have a proclivity to assume a spindle-like morphology which is a distinct feature of previously published images of endothelial cells. In vivo studies have demonstrated the incorporation of CD133+ EPCs into loci of angiogenesis in ischemic tissue. Ashahara and colleagues have shown that bone marrow-derived EPCs are released into peripheral circulation by ischemic stimulation in adult animal models. There are populations of stem cells in the bone marrow with the ability to differentiate also into cells with characteristics of cardiac muscle.
Given the favorable results from several animal studies, investigators have conducted clinical trials with CD133 stem cells in patients with cardiovascular disease one of them published recently (Klein et al. ,Steinhoff et. al and Pompilio et.al.). Patients who received the CD133 cells showed improved perfusion in areas where stem cells have been injected. This resulted in a significant increase in left ventricular ejection fraction, regional wall motion in the infarct zone, and a reduction in end systolic left ventricular volume. No side effects especially those related to inflammation or arrhythmias were observed in all clinical trials dealing with CD133+ stem cells.

11.2 CD133+ stem cell preparation

All procedures related to cell selection are done in a operating room, connected to a second operating room where the patients are treated surgically. The patient will undergo bone marrow aspiration from the iliac crest under general anesthesia using a technique to maximize the number of bone marrow-derivd cells. Approximately 200-250 ml of bone marrow aspirate will be harvested.
The selection of CD133+ cells from bone marrow is achieved by the following procedure:
CD133+ cells will be purified from the wasted bone marrow cells by labeling with the CliniMACS CD133 reagent (magnetic antibody beads) followed by automated sorting through

© Klein / Coordination Centre for Clinical Trials Duesseldorf

CONFIDENTIAL

the CE certified CliniMACS closed cell selection system. After incubating the cells with the CD133 Reagent in PBS/EDTA Buffer supplemented with 0.5% human serum albumin (HSA), the excess of unbound reagent is removed by washing with the PBS/EDTA Buffer supplemented with 0.5% HSA. During the following automated selection the unwanted cells are removed and, in the final step, the selected cells are eluted from the column by the means of the PBS/EDTA Buffer. The selected cells can be used immediately after analysis for injection into the patient. The device is non-invasive in all aspects that involve processing of the cellular harvest and is not connected to the patient at any time. The cell separation process is performed during the Bypass//TMLR surgery. Purified autologous CD133+ cells will be injected directly into the myocardium around the laser holes.

11.3 CliniMACS Technology

The CliniMACS® System is based on magnetic cell sorting. By using the specific antibody interaction with cell surface antigens, heterogeneous cell mixtures can be separated in a magnetic field using an immunomagnetic label specific for the cell type of interest. The cells to be isolated are specifically labeled with super-paramagnetic particles. The super-paramagnetic particles are small in size (about 50 nm in diameter) and are composed of iron oxide/hydroxide and dextran conjugated to monoclonal antibodies. These antigen specific magnetic particles can be used to target cell types of interest for enrichment or depletion.
Labeling is performed by incubating the cell population with the stem cell specific CD133 Reagent. After magnetic labeling, the cells are pumped through a high-gradient magnetic separation column as described below. The magnetically labeled cells are retained in the magnetized column while the unlabeled cells flow through. The retained cells are eluted by removing the magnetic field from the column, then washing the cells out and collecting them in a bag.

The CliniMACS® System is for the clinical scale separation of cells intended directly for therapeutic applications. The instrument will separate the CD133 cells in a fully automated process yielding a highly enriched population of targeted cells. The CliniMACS CD133 Reagent is composed of monoclonal antibody towards the CD133 antigen conjugated to magnetic particles. The CD133 antigen is expressed on hematopoietic stem and progenitor cells derived from peripheral blood, umbilical cord blood and bone marrow. CliniMACS enriched CD133+ stem cells have multiple potential clinical applications currently being evaluated for safety and feasibility, including stem cell transplants for tissue repair/regeneration (e.g. intramyocardial transplant in patients after myocardial infarction) as well as immuno-deficient patients with malignancies or immune disorders
The CliniMACS Instrument together with the sterile CliniMACS CD133 Reagent, the single-use, sterile disposable CliniMACS tubing set and the sterile, isotonic phosphate buffered, 1 mM EDTA, saline solution has been inspected and approved by TÜV product services with the CE Mark in 2001. The CliniMACS CD 133 reagent is classified as a medical device of Class III and the CliniMACS cell selection system, tubing set and buffer are medical products of Class II.

© Klein / Coordination Centre for Clinical Trials Duesseldorf

C O N F I D E N T I A L

12 Investigational Procedure

12.1. CABG surgery

12.1.1 Operative Procedure

The preoperative preparation will follow established standards and include the necessary ECG electrodes and Transesophageal Echocardiogramm (TEE), if the latter is thought necessary by the surgeon. Anaesthesia, opening of the chest and initiation of the cardiopulmonary bypass (ECC – extracorporal circulation) will be performed according to the local guidelines. Off-pump surgery is not in accordance with the protocol.
Intraoperatively, on observing the heart, the surgeon will assess the coronary artery anatomy of the heart and identify graftable, as well as non-graftable vessels and the region that may be treated with TMR. The inspection and determination of coronary artery anatomy, in conjunction with patient’s clinical condition (ability to withstand additional time on the pump) will be the final decisive factor to determine whether the patient can take part in the study. Thereafter, the arteries that can be bypassed will be grafted in the usual manner. The LASER procedure will be performed in the rewarming period of the heart. To achieve this the Laser should be prepared while the aortic anastomosis is performed. Thereafter TMR can be initiated on the non beating heart. TMR will be performed in the distribution of the non-bypassable vessel, avoiding overlap into the distribution of the bypassed vessels. The procedure is described in more detail below.

Afterwards the stem cell sample will be injected in a certain pattern around each laser channel.

The patient will be weaned from cardiopulmonary bypass after completion of TMR. Closure of surgery as well as post-operative care will be consistent with that following conventional CABG surgery, according to the guidelines set out below.

In order to enable the assessment of the surgical procedure and to break the "black box judgement" in studies on the effectiveness of surgical procedures. The surgeons will assess:

1. the difficulty of the operation (easy-intermediate-difficult) and
2. whether the operative procedure went according to plan.

© Klein / Coordination Centre for Clinical Trials Duesseldorf

C O N F I D E N T I A L

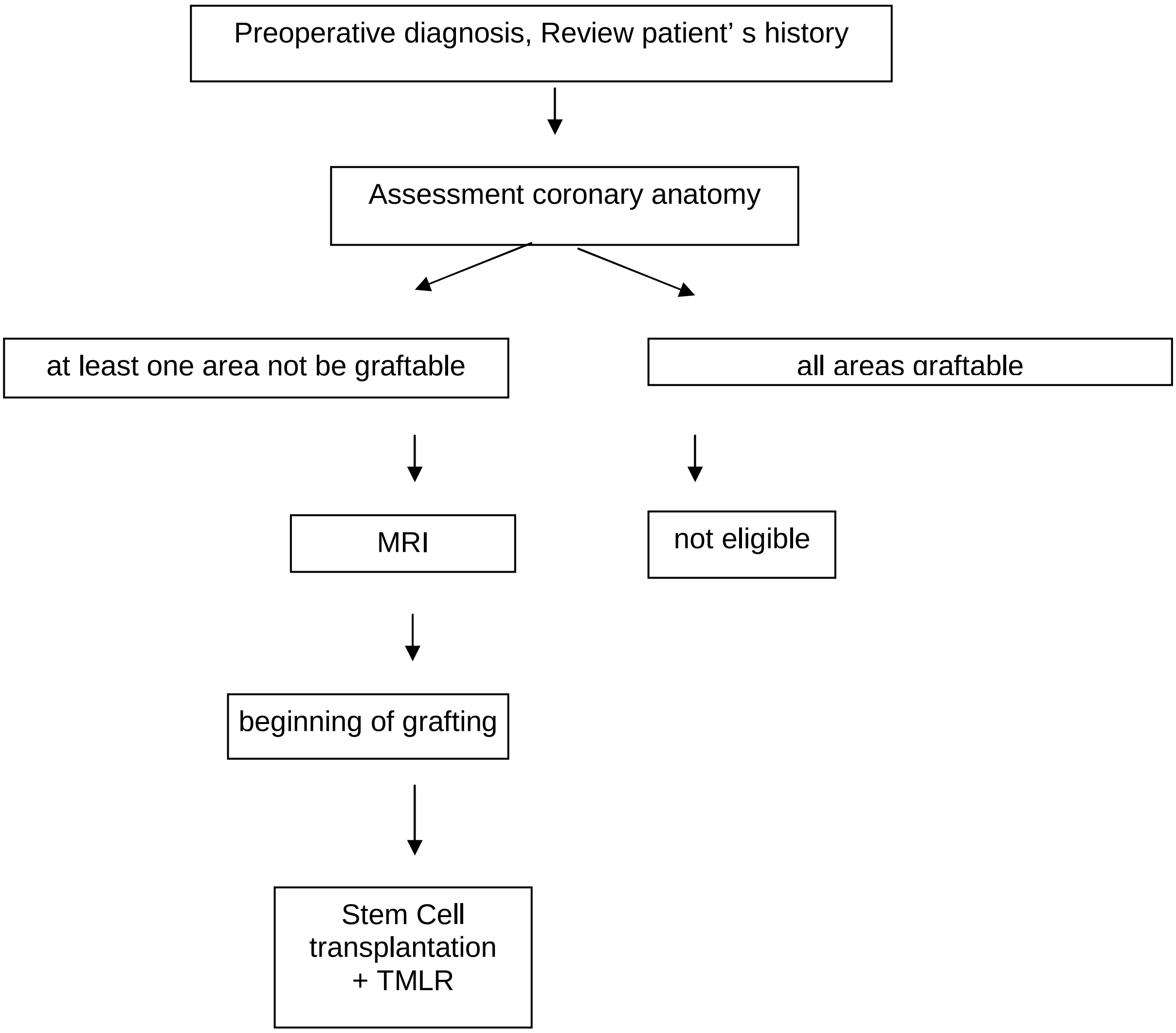

12.1.2 TMR and Stem Cell Transplantation

It is the surgeon's responsibility to be confident with the operational manual of the Laser device used (i.e. pre-trial technical training by the company) and to accept the clinical training by the principal investigators on site. ***No site should therefore begin the trial before the Principal/Coordinating investigators have been present on site to ensure the investigator is familiar with the TMR procedure.***

In order to assure a clinically relevant therapeutic intervention the area treated by TMR should be at least one of three free walls of the left ventricle. The free ventricular wall is described as the total of the anterior, posterior and posterior lateral wall of the left ventricle. The septum of the heart should not be treated by TMR, as this increases the risk of damage to the internal structures of the ventricle.

The TMR procedure cannot begin until the aortic clamp has been removed. The surgeon has to wait until the beginning of reperfusion. The surgeon has to assure that the left ventricle is sufficiently filled with blood to protect the posterior wall and mitral valve apparatus as well as

© Klein / Coordination Centre for Clinical Trials Duesseldorf

C O N F I D E N T I A L

the septum from injury by the Laser. If vent is used, venting has to be stopped at the beginning of the laser procedure. The actual TMR procedure has to be done before the heart has been weaned off the by-pass. As trigger the internal trigger of the laser has to be used at 100 beats/minute independent of the actual pulse frequency of the beating or fibrillating heart. The maximum energy suggested for the procedure is 25J. However, should the heart be covered by a layer of fat or have unusually thick walls, an energy of up to 40J may be required to achieve penetration of the walls; under these special conditions a higher energy than 25J is permissible.

Usually after TMR the bleeding is controlled by protamin. Generally manual compression will assure hemostasis. Infrequently, if excessive bleeding does occur, a single epicardial suture can be used.

Optimal spacing of the LASER holes is with 1 cm distance, for illustration of the treatment area, see below. The number of channels must be at least 10 holes per territory and will be documented. As a guideline it is suggested that the territory where TMR is applied should be divided according to the illustration. The Stem Cells must be injected with into the surrounding of the laser channel. The red marks show the location of the Stem Cell transplantation, the black ones the sites where the laser channels are made:

Suggestions:

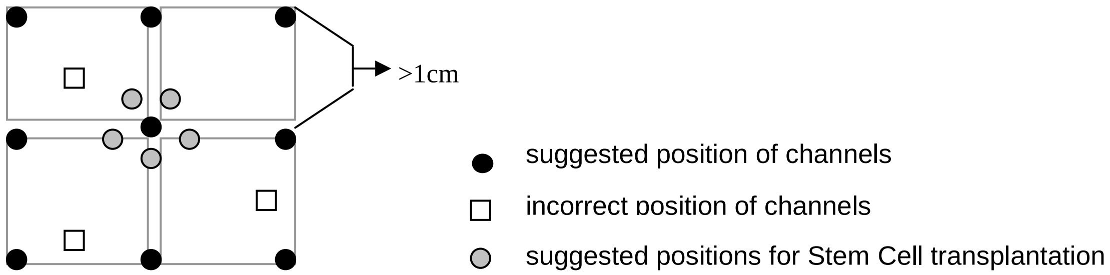

It is the surgeons responsibility to assure thorough understanding of the LASER treatment and the relevant precautions. To assure this competence the American Society For Laser Medicine And Surgery standards of practice should be followed. They can be found in the physician's instructions for the The Heart Laser^{TM} Co_2 TMR SYSTEM in the addendum of the protocol.

12.1.3 TEE (transesophageal endoscopy)

TEE has been considered an essential adjunct to TMR but surgeons experienced in the usage of TMR may not need this intraoperative help, in order to enable reliable assessment of successful penetration into the left ventricular cavity. Channels are marked by bubbles formed by photomolecular disruption of elemental gases by LASER energy in the LV cavity and by spurting of bright red blood from the drilled holes.

© Klein / Coordination Centre for Clinical Trials Duesseldorf

CONFIDENTIAL

12.1.4 Post-operative Care

After surgery post-operative cardiac status is assessed as follows:

1. circulation stability without inotropic drugs
2. as in 1 but dependent on low-dosage inotropic drugs
3. circulation stability but dependent on high dosage inotropic drugs or assisted circulation
4. low output syndrome in spite of high-dosage inotropic drugs with or without assisted circulation

High dosage inotropic drugs is defined as follows:
Single dose of:

dopamine	$\geq$	8 µg/kg/bodyweight
dobutamine	$\geq$	6 µg/kg/bodyweight
epinephrine	$\geq$	0,1 µg/kg/bodyweight
norepinephrine	$\geq$	0,1 µg/kg/bodyweight

or

Combination of cathecholaminic drugs (dopamine > 3µg/kg/bodyweight)

If the patient is in low cardiac output (as established by Swann Gans catheter reading and/or other intracardiac monitoring devices) then pharmacological support is instituted, followed by, if necessary, mechanical support, e.g. balloon pump or other mechanical devices. Weaning from the above should be according to local guidelines.

The principles of perioperative management include mechanical rather than inotropic support, reduction of perioperative pain and stress.

Because there is reduced ventricular compliance and a tendency towards myocardial ischaemia in response to stress, there is a definite need for higher left ventricular filling pressure observed within the first 24 hours postoperatively. Diuretics are given as early as possible. Mechanical support initiated prophylactically for low preoperative ejection fraction is continued until there is no clinical need.

Patients with unstable angina often require several additional days on the intensive care unit when they are weaned from their intravenous antianginals. Cardiac suppressants (ß-blockers, calcium channel blockers) are avoided during the first 48h, if in accordance with the local guidelines. Preoperative antianginals are restated as directed by pulse and blood pressure according to local guidelines. Myocardial ischaemia is aggressively treated with heparin, nitrates, and mechanical support. If intractable, salvage with percutaneous angioplasty is considered.

Low cardiac output may develop in the early postoperative period secondary to myocardial edema, recurrent ischaemia, tamponade or delayed chordal rupture. These adverse events should be assessed by echocardiography and appropriate therapy can be instituted. In addition to this, the amount of chest wall edema and air generally precludes adequate assessment with transthoracic echo. These patients are intolerant of any significant perioperative stress, therefor rapid identification of the etiology and institution of treatment is necessary.

© Klein / Coordination Centre for Clinical Trials Duesseldorf

C O N F I D E N T I A L

While discharge medication in general is the surgeons responsibility, minimum discharge medication for all patients must include ASS 75mg – 100mg OD or therapeutic equivalent in case of contraindications against ASS.

Duration of hospital stay is left to the treating surgeons but for the purpose of this trial the minimum duration of stay is 5 days on the surgical wards. Should the patient need to stay in hospital more than 14 days, this is considered a serious adverse event and the sponsor, represented by the principle coordinating investor need to be informed. However, hospitalisation as such is not considered a MACE. Should any other condition that constitutes a MACE be fulfilled also, e.g. stroke or myocardial infarction, these should, however, be considered MACE.

13 Assessment of Efficacy / Safety Data

13.1 Overview

The safety analysis will encompass all relevant data collected perioperatively during follow-up data. The analysis will be exploratory and take all adverse events, expected or unexpected, into account.

At the pre-study visit the principal investigators will assess the surgeons and their units. To assure surgery according to the given quality standards only named surgeons will perform surgery of study patients at each centre.

13.2 Screening of patients

Patients who are screened, but not enrolled will be recorded on the Patient Screening Forms, along with basis demographic parameters and reasons for non-enrolment. This does facilitate assessment of the external validity of the trial, because enrolled patients can be compared to those not enrolled.
Additionally, in order to evaluate the diagnostic precision of preoperative assessment the screening log will be quite relevant. It is anticipated that more patients will be screened and consented than actually recruited. This is due to intraoperative assessment of the patient's condition prior to study inclusion. Thus, the screening log will include tick boxes to evaluate the reason for non-recruitment as well as the preoperative strategy in order to establish the differences of pre-operative assessment of the disease and intraoperative assessment, if these exist.

© Klein / Coordination Centre for Clinical Trials Duesseldorf

C O N F I D E N T I A L

13.3 Pre-operative Assessments

The following assessments must be made prior to a patient's inclusion

- physical examination and history
- concomitant/cardiac medication
- Angina class assessment (CCS)
- Seattle Angina Questionnaire
- 12-lead ECG
- evaluation of pre-operative eligibility criteria
- Informed consent
- laboratory parameters (baseline and cardiac markers)
- Chest Xray

Within 1 month prior to surgery, the Echocardiography must have been performed:

- MRI or CT in case of contraindication
- Echocardiography
    - ejection fraction
    - mitral valve function
    - akinetic areas
    - initiation of special tape to be used for all follow-up visits

Within 3 months prior to surgery, the Angiogram must have been performed:

- Angiogram

13.4 Operative procedure

laboratory parameters

- laboratory parameters (cardiac markers)
- HAMA

documentation of treatment

- total time of procedure
- number, location and type of bypass graft
- area in which Stem Cells are transplanted, number of injections and the volume which has been injected in total
- area of TMR treatment
- number of TMR channels

patient safety documentation

- time on cardiopulmonary bypass pump
- major intra-operative adverse events & complications
- need of blood transfusion

- postoperative need of drug or medical support on bypass
- need for pacemaker
- post operative rhythm and use of antiarrhythmic drugs
- duration of mechanical support
- post-operative cardiac status

documentation of perioperative treatment

- time ventilated
- duration of ICU stay
- duration of hospital stay
- need of drug or mechanical support

14 Patient Follow-Up - Post-Operative & Pre-Discharge

It is understood that the surgeon and the treating cardiologist are free to increase their follow-up to the usual schedule and add investigations they feel are helpful. However, for the purpose of the trial the follow-up set out below constitutes the minimum necessary for adequate documentation of the patients progress and must be followed closely.

12, 24 & 48 Hours Post-operatively (plus minus 3 hours):

- laboratory parameters (baseline and cardiac markers)
- 12-lead ECG
- evaluation of complications & unanticipated Adverse Effects
- MACE
- physical examination

earliest Day 4 (day of discharge or the day before)

- MACE
- Concomitant/cardiac medications
- physical examination
- evaluation of complications & unanticipated Adverse Effects
- 12-lead ECG
- Chest Xray

NB: hospital stay longer than 14 days is considered SAE but not necessarily a MACE, if no other condition other constituting a MACE is the cause of this prolonged stay. The place of discharge to which the patient is transferred/discharged has to be documented for all patients.

<u>Month 3, 6 and 12 (+/- 14 days) follow-up:</u>

- laboratory parameters (baseline and cardiac markers)
- physical examination
- concomitant/cardiac medication
- Angina class assessment (CCS)

- Seattle Angina questionnaire
- MACE
- MRI or CT (only after 6 months)
- Echocardiography (within one month before or after follow-up)
- 12-lead ECG
- evaluation of complications & unanticipated Adverse Effects
- Chest Xray (12 months)

NB: A patient card is provided for the patients to inform other treating physicians of the trial and the need for follow-up. This is meant to facilitate the assessment of hospitalisation as well as the assessment of adverse effects. These need to be printed.

15 Medical Treatment of the patients after completion of the trial

Patients will be released in standard cardiological attendance.

© Klein / Coordination Centre for Clinical Trials Duesseldorf

C O N F I D E N T I A L

STUDY-TABLE	Screening/ Pre-operative Assessment	Operative Procedure	12 hrs post-surgery	24 hrs post-surgery	48 hrs post-surgery	pre-discharge (day 4 earliest)	Month 3	Month 6	Month 12
Informed consent	X								
evaluation of pre-op. eligibility criteria	X								
laboratory parameters	X[1]	X[1,2]	X[1]	X[1]	X[1]	X[1]	X[1]	X[1]	X[1]
general evaluation of complications		X	X	X	X	X	X	X	X
evaluation of AEs/SAEs		X	X	X	X	X	X	X	X
physical examination and history	X		X	X	X	X	X	X	X
concomitant/cardiac medication	X					X	X	X	X
MACE			X	X	X	X	X	X	X
MRI[3]	X[4]							X[4]	
Cardiac CT[3]	X[4]							X[4]	
Chest Xray	X					X			X
12 lead ECG	X		X	X	X	X	X	X	X
Echocardiography	X[4]						X[5]	X[5]	X[5]
Angiogram	X[6]								
Seattle Angina Questionnaire	X						X	X	X
Angina class assessment (CCS)	X						X	X	X
Documentation of treatment		X							
Documentation of patient safety		X							
Documentation of perioperative treatment		X							

[1] including baseline CK, CK-MB, CK-MB (Masse) Troponin I, Troponin T (except HAMA)
[2] only HAMA
[3] In case of contraindication against cardiac MRI the cardiac function is assessed by cardiac CT
[4] within 1 month prior to surgery
[5] within 1 month before or after follow-up
[6] within 3 months prior to surgery

16 Adverse Events

16.1 Adverse Event Monitoring

Patients will regularly be monitored for development of adverse events during the visits. The Sponsor, represented by the principle coordinating investigators will be timely informed about all serious adverse events.

16.2 Adverse Event Definitions

16.2.1 Adverse Event

An adverse event (AE) is any untoward medical occurrence in a subject. This does not imply that there is a relationship between the adverse event and the intervention under investigation. An AE can therefore be any unfavourable and unintended sign (including an abnormal laboratory finding, for example), symptom, or disease temporally associated with the investigational procedure, whether or not considered related to the investigational investigational procedure. For cardiac AEs the CTC-criteria (see supplement) are used.

All AEs will be assessed by the investigator for severity, using the following grading scale:

- Mild: usually transient requires no special treatment and does not interfere with patient’s daily activities.
- Moderate: produces a low level of inconvenience to the patient and may interfere with daily activities. These events are usually ameliorated by simple therapeutic measures
- Severe: interrupts daily activity and requires systemic drug therapy or other medical treatment

A pre-existing abnormal laboratory finding prior to administration of study intervention will not be classified as AE unless it is increasing in severity during the course of the study.

16.2.2 Serious Adverse Event

Events that pose a threat to a patient's life or functioning are considered “serious”.
A serious adverse event (SAE) is any untoward medical occurrence or effect that at any dose

- results in death
- is life-threatening
- requires hospitalisation or prolongation of existing inpatients’ hospitalisation
- results in persistent or significant disability or incapacity
- is a congenital anomaly or birth defect

© Klein / Coordination Centre for Clinical Trials Duesseldorf

C O N F I D E N T I A L

Medical and scientific judgement will be exercised in deciding whether expedited reporting is appropriate in other situations, such as important medical events that may not be immediately life-threatening or result in death or hospitalisation but may jeopardise the patient or may require intervention to prevent one of the other outcomes listed in the definition above. These will also usually be considered serious.
Life-threatening in the definition of a serious adverse event or serious adverse reaction refers to an event in which the subject was at risk of death at the time of event; it does not refer to an event which hypothetically might have caused death if it were more severe.
Hospitalisation is defined as at least one overnight stay in a hospital. Treatment on outpatient basis is not regarded as hospitalisation.
Should an AE be considered serious it must be additionally documented and reported on a separate SAE report form.

16.2.3 Potential Risks and Complications

The potential complications/risks associated with this protocol are as follows:

- intra-operative arrhythmia or generation of arrhythmic foci post-operatively
- ventricular aneurysm
- thromboembolic events (e.g. pulmonary embolism, stroke, myocardial infarction)
- damage to the chordae tendineae of the mitral valve intraoperatively, possibly resulting in acute mitral valve regurgitation
- additional time under anesthesia and on cardiopulmonary bypass
- haemorrhage
- infection
- death

16.2.4 Unexpected Adverse Event

Any adverse reaction, the nature, or severity of which is not consistent with the applicable information about the investigational procedure are regarded as unexpected.

16.2.5 Adverse Reaction

An adverse reaction of an investigational procedure is any untoward and unintended responses to an investigational procedure causally related to the procedure.

16.2.6 Suspected Unexpected Serious Adverse Reactions (SUSARs)

A suspected unexpected serious adverse reaction (SUSAR) is any adverse reaction, which is not consistent with the nature or severity listed in the applicable informations of the investigational procedure fulfilling the criteria of seriousness (cf. 16.2.2).

© Klein / Coordination Centre for Clinical Trials Duesseldorf

C O N F I D E N T I A L

16.3 Relationship of Adverse Event to Investigational Procedure

For each reported adverse event, the investigator must make an assessment of the relationship of the event to the investigational procedure.

The relationship to the investigational procedure should be assessed using the following definitions:

- **Certain**: A clinical event, including laboratory test abnormality, occurring in a plausible time relationship to the investigational procedure, and which cannot be explained by concurrent disease or other drugs or chemicals.
- **Probable**: A clinical event, including laboratory test abnormality, with a reasonable time sequence to administration investigational procedure, unlikely to be attributed to concurrent disease or other drugs or chemicals.
- **Possible**: A clinical event, including laboratory test abnormality, with a reasonable time sequence to investigational procedure, but which could also be explained by concurrent disease or other drugs or chemicals. Information on drug withdrawal may be lacking or unclear.
- **Unlikely**: A clinical event, including laboratory test abnormality, with a temporal relationship to investigational procedure which makes a causal relationship improbable, and in which other drugs, chemicals or underlying disease provide plausible explanations.
- **Not assessed**: A clinical event, including laboratory test abnormality, reported as an adverse reaction, which was not judged at the time of reporting, because e.g. more data is essential for a proper assessment or the additional data are under examination.
- **Unassessable**: A report suggesting an adverse reaction which cannot be judged because information is insufficient or contradictory, and which cannot be supplemented or verified.

16.4 Documentation of Adverse Events

All AEs will be documented in the CRF. AEs will be classified to be “serious” or “non-serious”. These categories define the documentation and reporting of the corresponding adverse event.

The AEs will be recorded on the appropriate CRF page, including:

- date of onset and resolution
- severity
- relationship to the investigational procedure (surgery, laser treatment or Stem Cells)
- serious or non-serious
- discontinuation of study medication

Each adverse event will be followed until resolution or through the last day of the study and the last visit.

© Klein / Coordination Centre for Clinical Trials Duesseldorf

Pre-existing diseases present prior to investigational procedure, will be documented as concomitant diseases as part of patient history in the CRF. Any disease newly occurring or increasing in severity during the course of the study will be documented as an AE.

16.5 Reporting of Serious Adverse Events

Each SAE regardless of causal relationship occurring in the course of the study has to be documented on the corresponding CRF page.
Investigators will inform the principle coordinating investigator within 24h about any serious adverse event.
Investigators will inform the principle coordinating investigator as soon as possible after knowledge about a pregnancy of a participant and its outcome.

The SAE-Form should be send by fax to the following address:

Prof. Michael Klein, MD
Department of Thoracic and Cardiovascular Surgery
Heinrich-Heine-Universityhospital Duesseldorf
Moorenstr. 5
40225 Duesseldorf
Fax +49-211-81-17612

All suspected adverse reactions related to an investigational procedure which occur in the concerned trial, and that are both unexpected and serious (SUSARs) are subject to expedited reporting.

All SAEs will be evaluated by the principal coordinating investigator whether they fulfil the criteria of seriousness and expectedness. The principal coordinating investigator will notify the ethics committee and the competent authority (Paul-Ehrlich-Institut, PEI) about all such SAEs.

16.6 Follow-up of patient sustaining AEs/SAEs

All AEs or SAEs will be, as described in the study protocol, well documented, categorised and according to its particular medical necessity and severity treated.
The follow-up of these patients is guaranteed throughout the examinations at follow-up visits at 3, 6, and 12 months.

© Klein / Coordination Centre for Clinical Trials Duesseldorf

CONFIDENTIAL

16.7 Notification of the Ethics Committee and Competent Authority

Any suspected unexpected serious adverse reaction will be reported to the competent authority and ethics committee.

- **Fatal or life-threatening SUSARs**
  The competent authority and the Ethics Committee will be notified about all fatal or life-threatening SUSARs as soon as possible but no later than 7 calendar days after the sponsor has first knowledge of the minimum criteria for expedited reporting.
  In each case relevant follow-up information will be sought and a report completed as soon as possible. It will be communicated to the competent authority and the Ethics Committee within an additional eight calendar days.

- **Non fatal and non life-threatening SUSARs**
  All other SUSARs will be reported to the competent authority and the Ethics Committee as soon as possible but no later than 15 calendar days after the sponsor has first knowledge of the minimum criteria for expedited reporting. Further relevant follow-up information will be given as soon as possible.

In case of incomplete information at the time of initial reporting, all the appropriate information for an adequate analysis of causality should be actively sought from the reporter or other available sources.

Within 15 days the sponsor informs the ethics committee and the competent authority about all circumstances that lead to re-evaluation of the risk-benefit ratio.

This includes:

- Individual reports on expected serious reaction with unexpected outcome,
- Increase of incidence of expected serious reactions, which is regarded clinically relevant,
- SUSARS which occurred after the affected person has left the trial,
- Events in context of the trial or development of the clinical investigational plan, which might impact the safety of affected persons.

16.8 Information of the Investigator

The sponsor informs the investigators about all SUSARs including all relevant additional information.
If new information becomes available that differs from the scientific information to the investigators that may interfere with the safety of patients in the trial or with the conduct of the study the sponsor will provide this information to all investigators.

© Klein / Coordination Centre for Clinical Trials Duesseldorf

C O N F I D E N T I A L

16.9 Annual safety reports

In addition to the expedited reporting, the sponsor will submit, once a year throughout the clinical trial or on request a safety report to the competent authority, the Ethics Committee of the concerned Member States and the Coordination Centre for Clinical Trials Duesseldorf, taking into account all new available safety information received during the reporting period. This global analysis should be the same for the competent authorities the Ethics Committee concerned and the Coordination Centre for Clinical Trials Duesseldorf.

16.9.1 Content of the annual safety report of a clinical trial

The periodic annual safety report covers the following aspects:

- A report on the subjects' safety in the concerned clinical trial,
- A line listing of all suspected serious adverse reactions (SAR), including all SUSARs, occurred in the concerned trial
- An aggregate summary tabulation of suspected SARs that occurred in the concerned trial.

16.9.2 Reporting time frame for annual safety report

The reporting time frame for annual reports starts with the date of the first authorisation of the concerned clinical trial by the competent authority. This date is designated as the cut off for data to be included in the annual safety report. The will submit annual reports within 60 days of the data lock point.

17 Duration of Study / End of Study

The study is planned for one year of recruitment and one year of follow-up for each participant. Totalling about 2 years, should recruitment be according to schedule. The end of study is defined at the time-point when the last patient has had his last visit (last patient out).

Criteria to finish the clinical trial:

- insufficient number of including patients
- negative risk-benefit-analysis
- latest scientific findings that negates the aims of the study

18 Statistics

18.1 Sample Size

The sample size for this pilot or feasibility study will be fixed at 40 patients treated in three centres. This number will give an approximated idea of the results that can be expected to be gained in a larger controlled clinical trial. The number of patients is the minimum to assure that the endpoints that may be relevant in a later clinical trial (i.e. the endpoints of this trial) can be

© Klein / Coordination Centre for Clinical Trials Duesseldorf

CONFIDENTIAL

evaluated for this follow-up trial. Because of the same incidence of male and female in CABG patients, an equal gender distribution is assumed.

18.2 Study sites

The trial is planned as a multicentre study. It is well established in surgery that the patients' outcome depends on the quality of the surgeon as well as on perioperative care, this is all the more relevant since stem cell transplantation is a relatively new therapy and the preperation as well as application of the autologous stem cells is sensitive and may be more successful in one centre than in another.

18.3 Statistical Analysis

Statistical analysis will be performed by the coordination centre of clinical trials (KKS). The data will be listed per centre and per patient. Descriptive statistics will be calculated.

19 Data Management

19.1 Administrative Aspects of the Investigational Study

All study data will be recorded onto Case Report Forms (CRFs) provided by the Coordination Centre for Clinical Trials. Data entered onto CRF pages will be typed or legibly printed using a black ball point pen. No freely handwritten documentation will be accepted. Although Case Report Form completion may be delegated to other study personnel, the Investigator(s) remain responsible for the accuracy and integrity of all data entered on CRFs.

Corrections to data already recorded on a Case Report Form will be made by drawing a single line through the erroneous data such that the original entry remains legible. The change must be initialed, dated and the reason for the change coded accordingly. Do not erase or white out errors. Where data are missing and a space on the CRF is blank, “ND” will be entered if the item was not done, “NA” if the item is not applicable or “UK” if unknown in respect of the individual case. If illegible or uncertain entries require clarification, the clarification may be printed above the original entry, initialed, dated and the reason for the change coded according to the code on the CRF.

19.2 Data Control Methods

In order to assure adequate control and provide study data that are consistent and of the highest quality, the following methods will be employed:

- Whenever possible, each clinical procedure (e.g. physical examination) for a particular patient will be conducted by the same person throughout the patient’s study participation.
- The appropriate specialist must review data generated automatically locally as appropriate; i.e. ECG interpretation must be reviewed and signed off by a cardiologist, if available.
- Photocopies of all diagnostic and operative reports, removing confidential patient identifiers, will be filed with the appropriate patient notes to assure Case Report Form verification.

© Klein / Coordination Centre for Clinical Trials Duesseldorf

Discharge letter should be collected from other hospitals for the duration of the trial, if they are available. It should be remembered that re-hospitalisation is a relevant endpoint.

19.3 Data Management

Data Management will be performed by the coordination centre of clinical trials (KKS). Completed CRFs will be sent to the KKS. Data will be entered centrally using the clinical data management system eResNet (eRT). This system has been validated according to industry standards.

20 Clinical Monitoring

This study will be monitored by representatives of the Coordination Centre for Clinical Trials.

Monitoring contains site visits (initiation visit, regular monitoring visits, close out visit) and regular telephone contacts will be made throughout the course of the study. During site visits, the monitor will review original patient records, device accountability and storage and general study procedures and discuss any problems with the Investigator(s). Case Report Forms (CRFs) will be verified against source documentation to verify accuracy.

To ensure that the study may be adequately monitored, the Investigator(s) or Study Co-ordinator will co-operative in providing to representatives of the Coordination Centre for Clinical Trials (KKS), all study documents (patient records and study files) and responding to inquiries that may arise as a result of the document review. KKS review of these documents will usually occur during a routine monitoring visit, but may also be required during a visit by a quality assurance auditor. KKS reserves the right to terminate the study if access to source documentation of work performed in this trial is denied to KKS medical representatives

21 Record Retention

General clinical and clinical study recordings of the patient, as:

- medical records
- Case Report Forms
- study-related correspondence and study reports
- Device Accountability records

will be retained for a minimum of fifteen years.

The Retain-Sample of the stem product will be stored according to GMP-directive for five years.

Patient relevant data regarding stem cell application while surgery, as:

- Patient-Identification
- All documents regarding the manufacturing of the stem cell product
- Date and time of stem cell application

will be retained regarding German Transfusionsgesetz § 14 for thirty years.

© Klein / Coordination Centre for Clinical Trials Duesseldorf

22 Ethics

The risks must be weighed against the potential benefits of the investigational protocol, which are as follows:

- increased perfusion to an area of the heart which otherwise could not be adequately treated
- improved left ventricular function
- reduction in anginal pain
- reduction in anti-anginal medication
- improved quality of life

This investigational plan has reduced the potential risks to the patient by:

- selecting eligibility criteria that will exclude those patients at highest risk of experiencing an anticipated adverse event;
- using routine post-operative echocardiograms to monitor for left ventricular or aneurysm; and
- using routine pre- and post-operative EKGs to detect arrhythmias

Declaration of Helsinki
The conduction of the trial is going to be in accordance with the last revision of the declaration of Helsinki (1996, Somerset West)

23 Ethics Committee/Institutional Review Board

The sponsors will submit the final protocol and proposed Informed Consent document to an Institutional Review Board (IRB). The clinical trial may not be initiated until appropriate documentation of IRB approval of the study protocol and the Informed Consent Form.

Modifications to the protocol will be submitted to the IRB for approval.

The Investigators will make appropriate and timely reports to the IRB as required by applicable government regulations and IRB policy. In addition to the progress reports, all known information regarding unanticipated adverse effects, whether observed at their clinical site or at another site participating in a clinical study with the investigational device, will be reported to the IRB. It is the Sponsor’s responsibility to inform the Investigators of unanticipated effects observed at other investigational sites.

It is the Investigators’ obligation to provide the Coordination Centre for Clinical Trials with copies of all study-related correspondence with the IRB in a timely fashion and to retain original within an appropriate filing system.

© Klein / Coordination Centre for Clinical Trials Duesseldorf

C O N F I D E N T I A L

24 Protocol Modification

Except in the event of a medical emergency or where it is necessary to protect the safety, rights or welfare of the study patient, any changes to the protocol will require written approval from Prof. Klein. Changes to the protocol will require that the protocol be formally amended. Protocol amendments will originate with Prof. Klein and will be agreed upon by the Investigators and approved of by the Institutional Review Board.

Administrative changes to the protocol such as a change that has no effect on the conduct of the study or risk to the patient will be documented in a memorandum originating at the Coordination Centre for Clinical Trials. The IRB must be notified of administrative changes, but formal approval is not required unless it is deemed necessary under the Standard IRB operating procedure of the participating institution.

Instances in which the Investigator(s) do not adhere to the protocol may result in “incomplete”, “unusable” or “not evaluable” data. Prof. Klein reserves the right to terminate the participation of any site should non-adherence to the clinical protocol occur or non-compliance is deemed excessive in nature.

Protocoll, patient information and consent forms are to be approved by the responsible ethics committees and recruitment cannot commence before approval has been given.

All necessary specifications and details of consent as well as the consent form are going to be part of the protocol submitted to the ethics committee for review.

25 Patient Informed Consent

Written informed consent must be obtained from each patient after adequate explanation of the aims, methods, objectives and potential hazards of the study will be obtained. And prior to the study screening evaluation. The investigator must also explain to the patients that they are completely free to refuse to enter the study or to withdraw from it at any time for any reason. The Informed Consent and Patient Information will be provided in German.

The patient, and the Investigator will sign the current IRB-approved version of the consent form and a signed copy of the consent form will be given to the patient. The data that consent was obtained will be recorded in the patient’s medical record.

Original, signed consent forms must be made available to the study monitor for inspection when requested

26 Insurance

Insurance is provided for patients taking part in the trial. In Germany this insurance is going to be in accordance with the “Allgemeinen Versicherungsbedingungen” of the Federal Republic of Germany. It will insure the patients against any injury caused by the trial to a maximum of 500000,- Euro per person. Insurance will be provided by the sponsor (delegated to the principal coordinating investigator) hired German insurance company. Patients will be informed about the insurance and their obligations in respect of the insurance coverage. The insurance policy and the insurance coverage can be found in the appendix E of this protocol.

© Klein / Coordination Centre for Clinical Trials Duesseldorf

C O N F I D E N T I A L

27 Publication Policy

The principal investigator will publish, together with all co-investigators, the clinical data and the results of the study in peer-reviewed journals. Authorships will be based on the overall contribution (i.e. recruitment, conduct, analysis) of the participating centres.
It is essential that the participating investigators exchange and discuss , prior to any publication or communication, any draft publication or communication made by the coordinating investigator. The investigators agree not to publish the results of the study pertaining to his/her centre prior to the publication of the overall study results.

28 List of Abbreviations

Abbreviation	Denotation
CABG	coronary artery bypass graft
ESRD	end stage renal disease
PCI	percutaneous coronary intervention
TIA	transient ischemic attack
QRS	measurement of the movement of electrical impulses through the lower heart chambers
Q-wave<br>II,aVF<br>I, aVL<br>V1-V6	 inferior leads (apex of the left ventricle)<br>lateral leads (lateral wall of the left ventricle)<br>anterior leads (frontal wall of the left ventricle)
AICD	automatic implantable cardioverter defibrillator
PRIND	prolonged reversible Ischemic Neurologic Deficit
OD	overdose
CK	creatine kinase
CK-MB	creatine kinase myocard
TMLR	Transmyocardial Laser Revascularization
CCS-Classification	Canadian Cardiovascular Society (CCS) Classification
SAQ	Seattle Angina Questionaire
MNC	mononuclear cells
BMC	bone marrow derived stem cells
LV	Left ventricular
TEE	Transesophargal ech
ECG	electrocardiogram
ULN	upper limit of normal range
HAMA	human anti-mouse-antibody

© Klein / Coordination Centre for Clinical Trials Duesseldorf

C O N F I D E N T I A L

29 References

1. Allen KB, Dowling RD, O'Neill WW et al. Comparison of transmyocardial revascularization with medical therapy in patients with refractory angina. New Engl J Med 1999; 341.
2. Fujii T, Yau TM, Li RK et al. Cell transplantation to prevent heart failure: a comparison of cell types. Ann Thorac Surg. 2003 Dec;76(6):2062-70; discussion 2070.
3. Frazier OH, March RJ, Hovarth KA et al. Transmyocardial revascularisation with a carbon dioxide LASER in patients with end-stage coronary artery disease. New Engl J Med 1999; 341: 1021-1028.
4. Hakuno D, Fukuda K, Makino S, et al. Bone marrow-derived regenerated cardiomyocytes (CMGlines) express functional adrenergic and muscarinic receptors. Circulation 1999; 100: II247-II256.
5. Karabatsch T, Schäper F, Hetzer R et al. Histological Findings after Transmyocardial LASER Re- vascularisation. J Card Surgery 1996;11 (326-31)
6. Kocher AA, Schuster MD, Wang et al. Neovascularisation of ischemic myocardium by human bone marrow derived angioblasts prevents cardiomyocyte apoptosis, reduces remodeling and improves cardiac function. Nat Med 2001;7 (4):430-436.
7. Loren J. Field et al., Differentiation and Long-Term Survival of C2C12 Myoblast Graft in Heart, J Clin Invest (1993.92:1548-1554) .
8. Loren J. Field et al.Formation of Nascent Intercalated Disks between Grafted Fetal Cardiomyocytes and Host Myocardium,Science, (1994, Vol 264, PP 98-100).
9. Makino et al, Cardiomyocyte can be generated from marrow stromal cells in vitro, J. Clin. Invest. 103: 697-705 (1999).
10. Orlic D, Kajstura J, Anversa P et al. Bone marrow cells regenerate infarcted myocardium. Nature 2001Apr 5; 410(6829):701-5.
11. Pesce M, Orlandi A, Capogrossi MC et al. Myoendothelial differentiation of human umbilical cord blood-derived stem cells in ischemic limb tissues. Circ Res. 2003 Sep 5;93(5):e51-62. Epub 2003 Aug 14.
12. Quirici N, Soligo D, Deliliers GL et al. Differentiation and expansion of endothelial cells from human bone marrow CD133(+) cells. Br J Haematol 2001 Oct; 115 (1):186-94.
13. Stamm C, Westphal B, Steinhoff G et al. Autologous bone-marrow stem-cell transplantation for myocardial regeneration. Lancet 2003 Jan 4;361 (9351):45-6.
14. Strauer BE, Brehm M, Wernet P et al. Repair of infarcted myocardium by autologous intracoronary mononuclear bone marrow cell transplantation in humans. Circulation 2002;106:1913-8.
15. Tang GH, Fedak PW, Li RK et al. Cell transplantation to improve ventricular function in the failing heart. Eur J Cardiothorac Surg. 2003 Jun;23(6):907-16. Review.
16. Wilke NM, Zenovich A, Muehling O, Jerosch-Herold M. Novel revascularisation therapies—TMLR and growth factor induced angiogenesis monitored with cardiac MRI. MAGMA 2000 Nov;11 (1-2):61-4.
17. Yamaguchi J, Kusano KF, Asahara T et al. Stromal cell-derived factor-1 effects on ex vivo expanded endothelial progenitor cell recruitment for ischemic neovascularization. Circulation. 2003 Mar 11;107(9):1322-8.
18. Yoo KJ, Li RK, Weisel RD, Mickle DA, Li G, Yau TM Autologous smooth muscle cell trans- plantation improved heart function in dilated cardiomyopathy. Ann Thorac Surg. 2000 Sep;70(3):859-65.

© Klein / Coordination Centre for Clinical Trials Duesseldorf

Supplements

MACE (Major adverse cardiac event)

MACE after surgery defined as either:

- Death
- MI (definition below)
- urgent or elective PCI (percutaneous coronary intervention) and repeat coronary surgery
- stroke/TIA
- any hospitalisation, according to cardiac symptoms

MI definition

**Timepoint**	**CK-MB criteria**	**ECG** (definition below)
predischarge after bypass surgery	not according to lab	Q-wave
associated with PCI	> 3 ULN	Q-wave, non-Q-wave
spontaneous MI	> 2 ULN	Q-wave, non-Q-wave

ECG changes of Q-wave MI:

Any QR wave in leads V1 through V3 ≥ 30 ms; abnormal Q wave in lead I, II, aVL, aVF or V4 through V6 in any two contiguous leads and at least 1 mm in depth.

In the absence of QRS confounders (e.g. bundle branch block, left ventricular hypertrophy, Wolff-Parkinson-White syndrome)

ECG changes of Non-Q wave

In the presence of a new CK/CK-MB increase, a ST segment elevation ≥ 1 mm, ST segment depression ≥ 1 mm, or T wave inversion ≥ 1 mm in at least two contiguous leads (i.e. II, III, avF; I, aVL, V5, V6, or V1-V5)

© Klein / Coordination Centre for Clinical Trials Duesseldorf

C O N F I D E N T I A L

Canadian Cardiovascular Society Grading Scale for Angina Pectoris

Class I	Ordinary physical activity does not cause angina. No angina occurs when walking or climbing stairs; angina does occur with strenuous or rapid prolonged exertion at work or recreation
Class II	Slight limitation of ordinary activity:Angina occurs when walking or climbing stairs rapidly; walking uphill; walking or stair climbing after meals, in the cold, in the wind, under emotional stress , or only during the first few hours after awakening; walking more than two blocks on the level and climbing more than one flight of ordinary stairs at normal pace and in normal conditions
Class III	Marked limitation of ordinary physical activity:Angina occurs when walking one or two blocks on the level and climbing one flight of stairs in normal conditions and at normal pace
Class IV A	Unstable angina resolved with intensified medical therapy and stabilised on oral medications
Class IV B	Unstable angina partly resolved on oral therapy, but symptoms return with minimal provocation
Class IV C	Unstable angina requiring acute care monitoring and parental or mechanical (e.g. intra-aortic balloon) therapy

Class I-III:
Cox J.; Naylor C.D.
The Canadian Cardiovasular Society Grading Scala for Angina Pectoris: Is it time for refinements ?
Ann Int Med 1992;117: 677 –683

Class IV A-C:
Cox JL, Nalor CD, Johnstone DE
Limitations of the Canadian Cardiovascular Society Classification of Angina Pectoris
Am J Cardiol 1994; 74: 276-277

© Klein / Coordination Centre for Clinical Trials Duesseldorf

C O N F I D E N T I A L

Laboratory parameters

**Hämatologie**
Leukocyten<br>Erythrocyten<br>Hämoglobin<br>Hämatokritt<br>MCV<br>MCH<br>MCHC<br>mittlere Ery. Verteilung
**Klinische Chemie**
Natrium<br>Kalium<br>Calcium<br>Creatinin<br>Harnstoff<br>Harnsäure<br>Totalprotein<br>Albumin im Serum<br>Bilirubin gesamt<br>direktes Bilirubin<br>GOT (37℃)<br>GPT 37C<br>y-GT (37°)<br>alk. Phosphatase (37℃)<br>Cholinesterade (37℃)<br>LDH (37℃)<br>CK gesamt (37℃)<br>CK-MB (37℃)<br>a-Amylase (37℃)<br>Lipase (37℃)<br>CRP<br>Glucose
**Haemostaseologie**
Quick<br>Intern. normalized ratio<br>APTT<br>APTT<br>TZ<br>TZ<br>Fibrinogen (QD)<br>Antithrombin
**Serologie**
Hepatitis B und D<br>HBs-Antigen
Hepatitis C<br>Anti-HCV-IgG<br>Anti-HIV1/2-Ig-G
**Kardiale Marker**
CK
CK-MB
CK-MB (Masse)
Troponin I
Troponin T
**Tumormarker/Hormone(Infektionsdiagnostik)**
T3 gesamt
T4 gesamt
TSH
**Immunologie**
HAMA

© Klein / Coordination Centre for Clinical Trials Duesseldorf

C O N F I D E N T I A L

CTC-Criteria documentation for cardiovascular adverse events:

CARDIOVASCULAR (ARRHYTHMIA)					
Adverse Event	**Grade**				
	**0**	**1**	**2**	**3**	**4**
Conduction abnormality/ Atrioventricular heart block	none	asymptomatic, not requiring treatment (e.g. Mobiz type I second-degree A V block, Wenckebach)	symptomatic, but not requiring treatment	symptomatic and requiring treatment (e.g. Mobitz type II second-degree A V block, third-degree A V block)	life-threatening (e.g., arrhythmia associated with CHF, hypotension, syncope, shock)
Nodal/junctional arrhythmia/dysrhythmia	non	asymptomatic, not requiring treatment	symptomatic, but not requiring treatment	symptomatic and requiring treatment	life-threatening (e.g., arrhythmia associated with CHF, hypotension, syncope, shock)
Palpitations	none	present	-	-	-
Note: Grade palpitations only in the absence of a documented arrhythmia					
Prolonged QTc interval (QTc>0.48 seconds)	none	asymptomatic, not requiring treatment	symptomatic, but not requiring treatment	symptomatic and requiring treatment	lifte-threatening (e.g. arrhythmia associated with CHF, hyptension, syncope, shock)
Sinus bradycardia	none	asymptomatic, not requiring treatment	symptomatic, but not requiring treatment	symptomatic and requiring treatment	life-threatening (e.g., arrhythmia associated with CHF, hypotension, syncope, shock)
Sinus tachycardia	none	asymptomatic, not requiring treatment	symptomatic, but not requiring treatment	symptomatic and requiring treatment of underlying cause	-
Supraventricular arrhythmias (SVT/atrial fibrillation/flutter)	none	asymptomatic, not requiring treatment	symptomatic, but not requiring treatment	symptomatic and requiring treatment	life-threatening (e.g., arrhythmia associated with CHF, hypotension, syncope, shock)
Syncope (fainting) is graded in the NEUROLOGY category					
Vasovagal episode	none	-	present without loss of consciousness	present with loss of consciousness	-
Ventricular arrhythmia (PVCs/bigeminy/trigeminy/ ventricular tachycardia)	none	asymptomatic, not requiring treatment	symptomatic, but not requiring treatment	symptomatic and requiring treatment	life-threatening (e.g., arrhythmia associated with CHF, hypotension, syncope, shock)
Cardiovascular/Arrhythmia – Other (Specify, ___________)	none	asymptomatic, not requiring treatment	symptomatic, but not requiring treatment	symptomatic and requiring treatment	life-threatening (e.g., arrhythmia associated with CHF, hypoten-sion, syncope, shock)

© Klein / Coordination Centre for Clinical Trials Duesseldorf

C O N F I D E N T I A L

CARDIOVASCULAR (GENERAL)					
Acute vascular leak syndrome	absent	-	symptomatic, but not requiring fluid support	respiratory compromise or requiring fluids	life-threatening; requiring pressor support and/or ventilatory support
Cardiac-ischemia(infarction	none	non-specific T-wave flattening or changes	asymptomatic, ST – and T – wave changes suggesting ischemia	angina without evidence of infarction	acute myocardial infarction
Cardiac left ventricular function	normal	asymptomatic decline of resting ejection fraction of ≥10% but <20% of baseline value; shortening fraction ≥24% but 30%	asymptomatic but resting ejection fraction below LLN for laboratory or decline of resting ejection fraction ≥20% of baseline value; <24%shortening fraction	CHF responsive to treatment	severe or refractory CHF or requiring intubation
Adverse Event	**Grade**				
	**0**	**1**	**2**	**3**	**4**
CNS cerebrovascular ischemia is graded in the NEUROLOGY category.					
Cardiac troponing I (CTnl)	normal	-	-	levels consistent with unstable angina as defined by the manufacturer	levels consistent with myocardinal infarction as defined by the manufacturer
Cardiac troponin T (cTnT)	normal	≥0.03-<0.05 ng/mL	≥0.05-<0.1 ng/mL	≥0.1-<0.2 ng/mL	≥0.2 ng/mL
Edema	none	asymptomatic, not requiring therapy	symptomatic, requiring therapy	symptomatic edema limiting function and unresponsive to therapy or requiring drug discontinuation	anasarca (severe generalized edema)
Hypertension	none	asymptomatic, transient increase by größer20mmHg (diastolic) or to >150/100* if previously WNL; not requiring treatment	recurrent or persistent or symptomatic increase by 20mmHg (diastolic) or to >150/100* if previously WNL; not requiring treatment	requiring therapy or more intensive therapy than previously	hypertensive crisis
*Note: For pediatric patients, use age and sex appropriate normal values >95th percentile ULN					
Hypotension	none	changes	requiring brief fluid replacement or other therapy but not hospitalization ; no physiologic conse-quences	requiring therapy and sustained medical attention, but re-solves without per-sisting physiologic consequences	shock (associated with academia and impairing vital organ function due to tissue hypoperfusion)
Also consider Syncope (fainting).<br>Notes: Angina or MI is graded as Cardiac-ischemia (infarction in the CARDIOVASCULAR (GENERAL) category<br>*For pediatric patients, systolic BP 65 mmHg or less in infants up to 1 year old and 70 mmHg or less in children older than 1 year of age, use two successive or three measurements in 24 hours*					
Myocarditis	none	-	-	CHF responsive to treatment	severe of refractory CHF
Operative injury of vein/artery	none	primary suture repair for injury, but not requiring transfusion	primary suture repair for injury, requiring transfusion	vascular occlusion requiring surgery or bypass for injury	myocardial infarction; resection of organ (e.g. bowel, limb)
Percardial effusion/pericarditis	none	asymptomatic effusion, not requiring treatment	pericarditis (rub. ECG changes, and/or chest pain)	with physiologic consequences	tamponade (drainage or pericardial window required)
Peripheral arterial ischemia	none	-	brief episode of ischemia managed non-surgically and without permanent deficit	requiring surgical intervention	life-threatening or with permanent functional deficit (e.g., amputation)
Phlebitis (superficial)	none	-	present	-	-
Notes: Injection site reaction is graded in the DERMATOLOGY7skin category.<br>Thrombosis/embolism is graded in the CARDIOVASCULAR (GENERAL) category.					
Syncope (fainting) is graded in the NEUROLOGY category.					
Thrombosis/embolism	none	-	deep vein thrombosis, not requiring	deep vein thrombosis, requiring	embolic event including

© Klein / Coordination Centre for Clinical Trials Duesseldorf

			anticoagulant	anticoagulant therapy	pulmonary embolism
Vein/artery operative injury is graded as Operative injury of vein/artery in the CARDIOVASCULAR (GENERAL) category					
Visceral arterial ischemia (non-myocardial)	none	-	brief episode of ischemia managed non-surgically and without permenent deficit	requiring surgical intervention	life-threatening or with permanent functional deficit (e.g., resection of ileum)
Cardiovascular/ General – Other (Specify, ____________)	none	mild	moderate	severe	life-threatening or disabling

COAGULATION					
Note: See the HEMORRHAGE category for grading the severity of bleeding events.					
DIC (disseminated intravascular coaglulation)	absent	-	-	laboratory findings present with <u>no</u> bleeding	laboratory findings <u>and</u> bleeding
Also sonsider Platelets.<br>Note: Must have increased fibrin split products or D-dimer in order to grade as DIC.					
**Adverse Event**	***Grade***				
	**0**	**1**	**2**	**3**	**4**
Fibrinogen	WNL	≥ 0.75-<1.0 x LLN	≥0.5-<0.75 x LLN	≥0.25-<0.5 x LLN	<0.25 x LLN
Partial thromboplastin time (PTT)	WNL	>ULN-≤1.5 x ULN	>1.5 ≤ 2x ULN	>2x ULN	-
Phlebitis is graded in the CARDIOVASCULAR (GENERAL) category.					
Prothrombin time (PT)	WNL	>ULN - ≤1.5 x ULN	<1.5-≤2x ULN	>2x ULN	-
Thrombosis/embolism is graded in the CARDIOVASCULAR (GENERAL) category.					
Thrombotic microangiopathy (e.g. thrombotic thrombocytopenic purpura/TTP or hemolytic uremic syndrome/HUS)	absent	-	-	laboratory findings present withut clini-cal consequences	laboratory findings and clinical conse-quences, (e.g., CNS hemorrhage/ bleeding or thrombosis/ embolism or renal failure) requiring therapeutic intervention
For BMT studies, if specified in the protocol	-	evidence of RBC destruction (schistocytosis) without clinical consequences	evidence of RBC destruction with elevated creatinine (≤3x ULN)	evidence of RBC destruction with creatinine (>3x ULN) no requiring dialysis	evidence of RBC destruction with renal failure requi-ing dialysis and/or encephalopat hy
Also consider Hemoglobin, Platelets, Creatinine.<br>Note: Must have microangiopathic changes on blood smear (e.g. schistocyted, helmet cells, red cell gragments)					
Coagulation – Other (Specify, ___________)	none	mild	moderate	severe	life-threatening or disabling

© Klein / Coordination Centre for Clinical Trials Duesseldorf

C O N F I D E N T I A L

LASER Treatment

The Heart Laser™ Co_2 TMR SYSTEM

Physician's Information for Use

Device description

The Heart Laser™CO_2 TMR System (The Heart Laser System) is a 1000 watt, fast axial flow, CO_2 laser, operated in a pulsed-only mode, producing pulses of 10 to 99 milliseconds at 8 to 80 joules. The energy is delivered directly to a heart through a seven mirror articulated arm terminated with a 125 millimeter focal hand piece producing an approximately 1 mm diameter hole. The CO_2 laser beam is combined with a helium-neon (visible) laser beam as it exists the laser tube to facilitate aiming.

The Heart Laser System is a pulsed, ECG synchronized 10,6 µm CO_2 laser intended for use in transmyocardial revascularization. The 1000 watt laser is set to deliver a maximum power of 800 watts in pulses to 99 msec long at energies of 8 to 80 joules. Laser energy is delivered to the tissue through an articulated arm terminated with a single use, sterile, handpiece. The laser housing contains all systems components, including the laser head, cooling system, power supply, and computer. All laser functions are controlled from the computer touch screen. The computer interfaces directly with the system H/P Model 78352C ECG monitor to trigger laser actuation at the peak of the R-wave. Laser pulse and ECG signals are displayed on the touch screen to allow the operator to see the timing of the laser pulse in relation to the R-wave. The sterile disposable TMR kit contains straight and right-angle handpieces to access different sections of the myocardium.

Indications for use

Transmyocardial revascularization (TMR) with The Heart Laser System is indicated for the treatment of patients with stable angina (Canadian Cardiovascular Society class 3 or 4) refractory to medical treatment and secondary to objectively demonstrated coronary artery atherosclerosis not amenable to direct coronary revascularization.

Warnings and Precautions

UNSTABLE ANGINA WAS ASSOCIATED WITH 22% PERI-OPERATIVE MORTALITY COMPARED TO 1% IN PATIENTS WITH STABLE ANGINA.

The randomized clinical study involving 151 TMR surgeries, patients were classified as stable or unstable angina (requiring IV anti-anginal medication) based on the two weeks preceding surgery. Peri-operative mortality (surgery + 30 days thereafter) was 1% (1/102) in the absence of unstable angina compared to 22% (11/49) when the patient suffered from unstable angina.
Right ventricular wall, septal ischemia – TMR with The Heart Laser System should not be used on patients with myocardial ischemia limited to the right ventricular wall and/or interventricular septum due to access limitations with TMR.
Laser pulse timing – The laser pulse to be timed at the peak of the R-wave. Do not operate The Heart Laser without proper placement of ECG electrodes.

© Klein / Coordination Centre for Clinical Trials Duesseldorf

C O N F I D E N T I A L

Explosions of fire hazard – Do not operate The Heart Laser in the presence of flammable gases, anesthetics, cleaning agents, combustible materials, or other volatile substances. Explosions of fire can result.

- Combustible or flammable materials (for example surgical drapes, gowns or gauze) in the surgical field may be ignited by CO_2 radiation unless they are kept wet or moistened.
- Surround the surgical field with wet towels or wet gauze.
- Modify all other flammable materials to make them fire-retardant (for example flame resistant surgical drapes and gowns).
- Minimize oxygen as oxygen increases the combustibility of materials exposed to CO_2laser radiation.
- Do not use plastic or rubber endotracheal tubes as the are highly flammable. Use firepool endotracheal tubes or protect the endotracheal tube from laser energy.
- Laser radiation – The Heart Laser System is a Class IV laser product.
- Avoid exposure to laser radiation at all times during the installation and operation of the laser as direct or reflected radiation may damage skin or eyes.
- DO NOT LOOK DIRECTLY INTO THE CO_2 LASER BEAM (not visible) or the helium – non laser aiming beam as either can cause permanent ocular damage.
- Protect the patient's eyes by covering them with wet gauze.
- -All operating room personnel must wear protective eyewear with a minimum optical density of 5 at a wavelength of 10.6 µm when The Heart Laser System is in use.
- Do not use shiny metallic surfaces within the operative field which may reflect the laser beam. Use instruments with a dull, anodised, or ebonized finish near the laser beam.

Physician Training

The Heart Laser should only be used by properly trained surgeons (see Section OPERATOR TRAINING).

Handling and Sterilization

- The TMR kit is for single use only. Do not re-sterilize or reuse.
- Inspect sealed sterile package before opening. If seal is broken, contents may not be sterile and may cause infection in the patient.
- After use, handle and dispose of TMR kit as appropriate for a biohazard.
- Clean exterior panels of laser of bio-contaminants following surgery
- Use only medical grade CO2 for the purge gas to prevent possible bio-contamination.

Precautions during TMR

- Transmural penetration by the CO_2laser should be confirmed by transesophageal echocardiography (see Section 6 CLINICAL STUDIES).
-

Packaging

The Heart Laser Systems consists of the laser unit and the sterile, disposable, TMR kit.

- The Heart Laser is initially installed in the Hospital by PLC personnel.
- The single-use TMR kits are supplied for every TMR case. Sterility may be compromised if the package is opened or damaged.
-

Storage

The storage life of the TMR kits is two years from date of sterilization.

Accessories

The TMR kit includes: Straight Handpiece (1); Right Handpiece (1), Laser Arm Drape (1), Heart Diagramm (2); Non-toxic marker (1); Mirror Swab (30); Plastic Trays (3).

Operator's Manual

Operating instruction for the laser unit is contained in the Heart Laser TMR System Operator's Manual which includes seven sections describing operator safety, a detailed device description, unpacking and installation procedures, system operation, accessories, and preventive maintenance. It also contains five appendices which describe the energy meter calibration procedure, the warranty, a bibliography of articles relevant to TMR, a list of sales/services contacts, and Physician's information for use.
It is essential that the Operator's Manual, especially those parts dealing with laser safety be read and understood before operating, maintaining and servicing this system. Failure to operate The Heart Laser System in accordance with the Operator's Manual may result in serious injury.

Operator Training

Federal law restricts the use of this device to practioners who have been trained in laser heart surgery including laser system calibration and operation. Operator training for use of The Heart Laser System must include training in the use of the laser system and sterile disposable kit as well as appropriate clinical training.

Laser Training

The American Society For Laser Medicine And Surgery offers the following Standards of Practice for the Use of Lasers in Medicine and Surgery:
Hospital privileges are, and must remain, the responsibility of the hospital governing board. Those requesting privileges to use lasers shall meet all the standards of the hospital with regard to board certification, board eligibility, special training, ethical character, good standing, judgement, indications for application, etc. In addition, the following laser training and experience are recommended.

- The individual should review the pertinent literature and audiovisual aids, and should attend laser training courses devoted audiovisual aids, and should attend laser training courses devoted to teaching of laser principles and safety. These courses should include basic laser physics, laser-tissue interactions, discussion of the clinical specialty field, and hands-on experience with lasers.
- The individual should have spent time with an experienced operator in the specialty area involved. It is essential that the individual see and document actual clinical applications of the laser in the outpatient or hospital setting as appropriate to the procedures in which the training is conducted.
- The individual should work closely with the biomedical engineering personnel.

© Klein / Coordination Centre for Clinical Trials Duesseldorf

C O N F I D E N T I A L

Clinical Training

Use of The Heart Laser System should only be undertaken by personnel trained in accordance with the PLC Medical Systems Training – Continuing Education Program. Comprehensive training program which includes:

- Surgical/clinical training for surgeons at Regional Training Centres, while the hospital nursing staff will receive on – site in-service training. For the initial clinical cases, new Heart Laser users will receive clinical support for patient selection and for TMR surgeries. Finally, PLC staff will conduct early follow-up visits to maximize the likelihood of positive initial clinical experience with TMR using The Heart Laser.
- An extensive continuing education program for existing clinical sites: This program will continuously improve the quality of TMR care through regular on – site-in-service training and ongoing communications with Heart Laser users.
- A complete emergency clinical support program: PLC staff will work with the TMR Advisory Board, which will be composed of 5 experienced TMR users, to respond to any TMR related requests for emergency assistance.

Further information about training can be obtained from your PLC Medical Systems. Inc. representative at +1-800-232-8422.

Mechanism of Action

The mechanism(s) whereby TMR relieves angina is not known. In addition to possible contribution of placebo effect, current theories include:

- Increased perfusion of myocardium via the channels created;
- Increased collaterization via angiogenesis,
- Symptom reduction resulting from disruption of pain fiber function;
- Possible microinfarcts to the myocardium.

Text supplied by PLC as found above

ECG-Monitor internal Trigger

Steps how to set up for the internal ECG trigger:

1. Switch off the ECG-Monitor and <u>wait</u> for 20 to 30 seconds
2. After 20 to 30 seconds push the **Cal Mark**- and the **Silence Reset** bottom and hold both bottoms pressed in while you switch on the ECG-Monitor at the same time. Continue holding both bottoms down!
3. Release both bottoms when the test trigger signal appears on the ECG-Monitor screen.
4. You should see now trigger pulse on the screen (Similar to the sketch on the right)

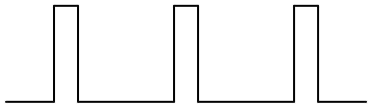

(at the beginning it is a little tricky because you have to hold the bottoms **Cal Mark** and **Silence Reset** pushed down together)

© Klein / Coordination Centre for Clinical Trials Duesseldorf

C O N F I D E N T I A L

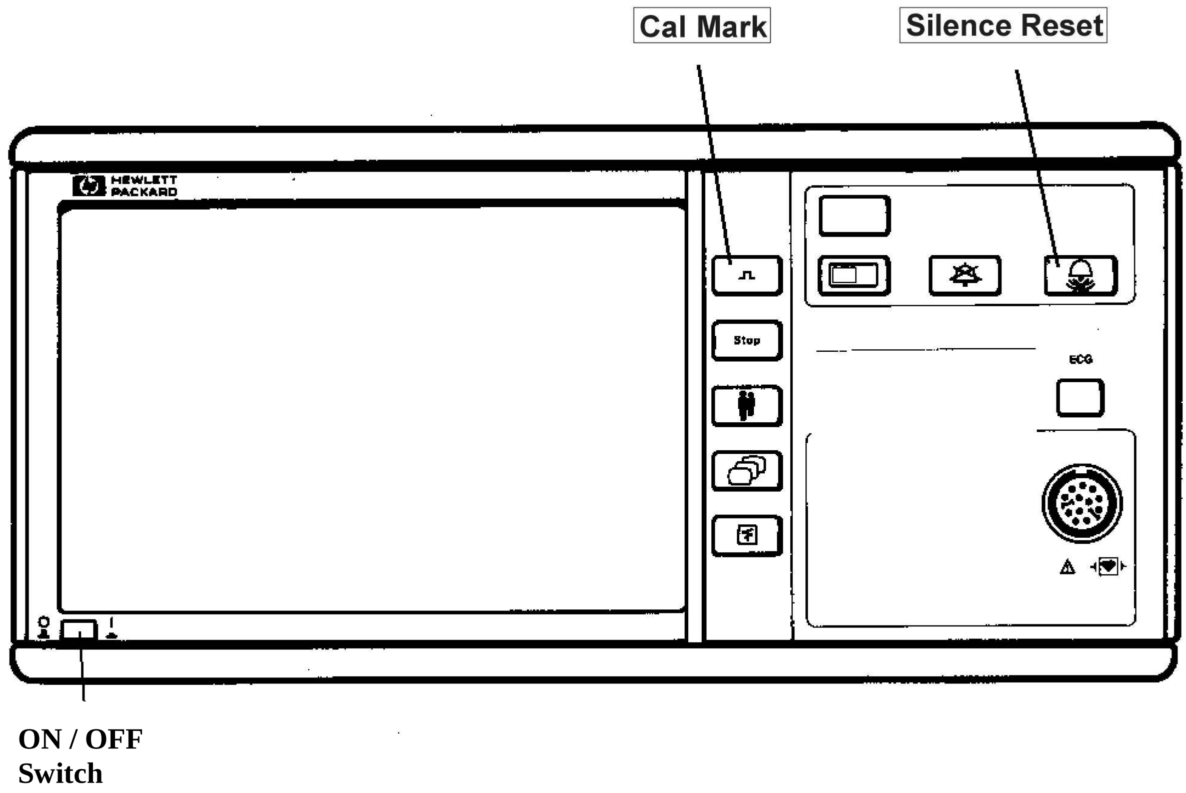

© Klein / Coordination Centre for Clinical Trials Duesseldorf

C O N F I D E N T I A L
