# Supplementary material for: Correction: Laser-Supported CD133+ Cell Therapy in Patients with Ischemic Cardiomyopathy: Initial Results from a Prospective Phase I Multicenter Trial
Source: PLoS One. 2014 Aug 25;9(8):e107082. doi: 10.1371/journal.pone.0107082 (PMC4143337; doi:10.1371/journal.pone.0107082)
Supplement: File S2 — Republished, corrected article. (PDF) [file pone.0107082.s002.pdf]

OPEN ACCESS Freely available online

PLOS | ONE

Laser-Supported CD133+ Cell Therapy in Patients with Ischemic Cardiomyopathy: Initial Results from a Prospective Phase I Multicenter Trial

Alexander Assmann[1,2,3,4]☯, Michael Heke[1,2]☯, Patric Kröpil[5], Lena Ptok[1], Dieter Hafner[6], Christian Ohmann[7], Andreas Martens[8], Antje Karluß[9], Maximilian Y. Emmert[10], Ingo Kutschka[8], Hans-Hinrich Sievers[9], Hans-Michael Klein[1]*

1 Department of Cardiovascular Surgery, Heinrich Heine University, Medical Faculty, Duesseldorf, Germany, **2** Research Group for Experimental Surgery, Heinrich Heine University, Medical Faculty, Duesseldorf, Germany, **3** Center for Biomedical Engineering, Department of Medicine, Brigham and Women's Hospital, Harvard Medical School, Boston, Massachusetts, United States of America, **4** Harvard-MIT Division of Health Sciences and Technology, Massachusetts Institute of Technology, Cambridge, Massachusetts, United States of America, **5** Department of Diagnostic and Interventional Radiology, Heinrich Heine University, Medical Faculty, Duesseldorf, Germany, **6** Institute of Pharmacology and Clinical Pharmacology, Heinrich Heine University, Medical Faculty, Duesseldorf, Germany, **7** Coordination Centre for Clinical Trials, Heinrich Heine University, Medical Faculty, Duesseldorf, Germany, **8** Clinic for Cardiothoracic, Transplantation and Vascular Surgery, Hannover Medical School, Hannover, Germany, **9** Department of Cardiac and Thoracic Vascular Surgery, University Hospital of Schleswig-Holstein, Luebeck, Germany, **10** Clinic for Cardiac and Vascular Surgery, University Hospital Zurich, Zurich, Switzerland

Abstract

Objectives: This study evaluates the safety, principal feasibility and restoration potential of laser-supported CD133+ intramyocardial cell transplantation in patients with ischemic cardiomyopathy.

Methods: Forty-two patients with severe ischemic cardiomyopathy (left ventricular ejection fraction (LVEF) $>$15% and $<$ 35%) were included in this prospective multicenter phase I trial. They underwent coronary artery bypass grafting (CABG) with subsequent transepicardial low-energy laser treatment and autologous CD133+ cell transplantation, and were followed up for 12 months. To evaluate segmental myocardial contractility as well as perfusion and to identify the areas of scar tissue, cardiac MRI was performed at 6 months and compared to the preoperative baseline. In addition, clinical assessment comprising of CCS scoring, blood and physical examination was performed at 3, 6 and 12 months, respectively.

Results: Intraoperative cell isolation resulted in a mean cell count of $9.7 \pm 1.2 \times 10^6$. Laser treatment and subsequent CD133+ cell therapy were successfully and safely carried out in all patients and no procedure-related complications occurred. At 6 months, the LVEF was significantly increased (29.7±1.9% versus 24.6±1.5% with $p = 0.004$). In addition, freedom from angina was achieved, and quality of life significantly improved after therapy ($p < 0.0001$). Interestingly, an extended area of transmural delayed enhancement ($>$3 myocardial segments) determined in the preoperative MRI was inversely correlated with a LVEF increase after laser-supported cell therapy ($p = 0.024$).

Conclusions: This multicenter trial demonstrates that laser-supported CD133+ cell transplantation is safe and feasible in patients with ischemic cardiomyopathy undergoing CABG, and in most cases, it appears to significantly improve the myocardial function. Importantly, our data show that the beneficial effect was significantly related to the extent of transmural delayed enhancement, suggesting that MRI-guided selection of patients is mandatory to ensure the effectiveness of the therapy.

Trial Registration: EudraCT 2005-004051-35) Controlled-Trials.com ISRCTN49998633

Citation: Assmann A, Heke M, Kröpil P, Ptok L, Hafner D, et al. (2014) Laser-Supported CD133+ Cell Therapy in Patients with Ischemic Cardiomyopathy: Initial Results from a Prospective Phase I Multicenter Trial. PLoS ONE 9(7): e101449. doi:10.1371/journal.pone.0101449

Editor: Giacomo Frati, Sapienza University of Rome, Italy

Received February 26, 2014; **Accepted** June 3, 2014; **Published** July 7, 2014

Copyright: © 2014 Assmann et al. This is an open-access article distributed under the terms of the Creative Commons Attribution License, which permits unrestricted use, distribution, and reproduction in any medium, provided the original author and source are credited.

Funding: This work was supported by the study sponsors PLC Medical Systems Inc. (Milford, USA) and Miltenyi Biotech Inc. (Bergisch Gladbach, Germany). The funders had no role in study design, data collection and analysis, decision to publish, or preparation of the manuscript. Moreover, this funding situation does not alter the authors' adherence to PLOS ONE policies on sharing data and materials.

Competing Interests: The authors have declared that no competing interests exist.

* Email: kleinhm@med.uni-duesseldorf.de

☯ These authors contributed equally to this work.

Introduction

While human myocardium cannot restore appropriate function after a significant loss of cardiomyocytes due to ischemic injury [1], all established therapeutic strategies, except heart transplantation with limited organ availability, focus on preventing further ischemia-induced myocardial damage. Therefore, alternative options for patients with severe ischemic cardiomyopathy are

Table 1. Study inclusion and exclusion criteria.

**Inclusion criteria**
>18 years (male or female gender)
Presence of at least two vessel coronary artery disease, with at least one vessel that is not amenable to CABG (according to the angiogram). This vessel must serve an area of viable myocardium
Global left ventricular ejection fraction >15% and <35%
Area of interest defined as part of free left ventricular wall with reduced contractility as preoperatively shown either in ventriculography and/or MRI/CT and/or echocardiography
Demonstration of reduced perfusion in the area of interest by cardiac MRI/CT
Signed informed consent
**Exclusion criteria**
Any condition that, according to the treating physician, prevents successful cell collection or application (e.g. systemic infection, puncture for cell collection impossible)
Any condition that may adversely affect the bone marrow (such as malignancy or prior irradiation to the pelvic bone)
Emergency or salvage operation defined as within 48 hours of diagnosis
Need of additional heart surgery (e.g. valve replacement)
Previous heart surgery within 6 months (excluding implantation of a pacemaker)
Mitral valve insufficiency >grade II
Evidence of left ventricular thrombus
Increased CK (>three times normal) in patients with unstable angina
End-stage renal disease defined as serum creatinine level >3.5 mg/dl, or renal replacement therapy
Concurrent active chemotherapy for cancer
Life expectancy ≤2 years
Clinically active infection at the time of operation
Hepatitis infection
HIV infection
Anemia
Platelet count lower than 100/nl
Sensitivity or incompatibility against used drugs or excipients (e.g. heparin, aspirin)
Pregnancy
History of ventricular arrhythmia, not controlled by medication and/or AICD (Automatic Implantable Cardioverter Defibrillator) required
History of symptomatic carotid disease (e.g. any TIA, PRIND, stroke) within the last 3 months prior to study intervention
History of bleeding diathesis
History of disseminated intravascular coagulation
Participation in other clinical trials (at least for the last 30 days)
Not able to attend follow-up as specified in the protocol
No informed consent

doi:10.1371/journal.pone.0101449.t001

required. Cardiac cell therapy has been repeatedly suggested as a promising strategy to improve the myocardial function in patients with chronic cardiac ischemia [2–4], and currently, numerous cell types are under evaluation for their regenerative potential [1].

Bone marrow cells (BMCs) represent the most frequently used cell type for cardiac cell therapy [5]. They can be harvested and up-scaled easily, and their clinical use evokes no legal or ethical issues. In this regard, CD133+ cells, a subpopulation of adult hematopoietic progenitor cells, are a well-established cell source and have been proven to beneficially affect chronic cardiac ischemia [6].

In parallel, the concept of transmyocardial laser revascularization (TMLR) has been shown to induce angiogenesis in a large animal model of chronic myocardial ischemia [7]. In order to create a supportive environment before intramyocardial cell transplantation, we recently combined the concept of TMLR and subsequent cell therapy in patients with end-stage coronary heart disease. This therapeutic approach resulted in remarkably increased wall contraction at the sites of TMLR and cell injection [8]. Therefore, we hypothesized that a combined approach may exert beneficial effects on patients with severe chronic myocardial ischemia. Based on these results, we modified and refined laser treatment to what we call 'endogenous laser-induced ventricular enhancement' (ELIVE). This novel approach is characterized by applying lower energies to specific areas in the heart pre-assessed by magnetic resonance imaging (MRI) for the purpose of inducing a supportive environment for the transplanted cells.

The present multicenter trial has been initiated to assess the safety and feasibility of CD133+ cell transplantation following coronary artery bypass grafting (CABG) and ELIVE in patients with ischemic cardiomyopathy. Moreover, the potential of this combined cell therapy approach to improve the myocardial

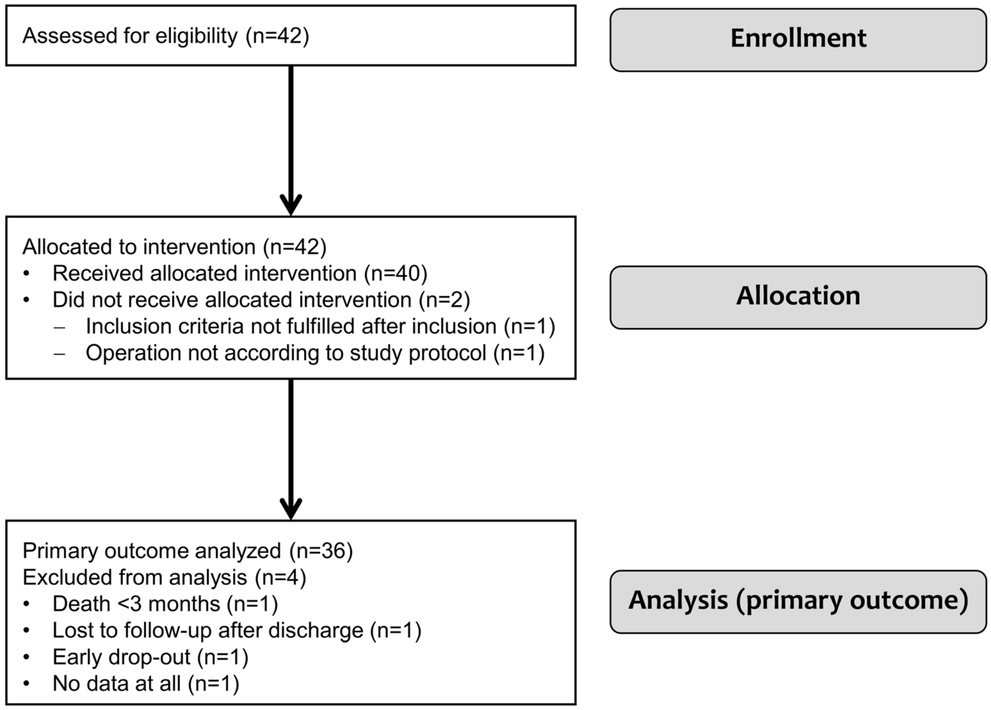

Figure 1. CONSORT (Consolidated Standards of Reporting Trials) flow diagram presenting the enrollment, intervention allocation, and data analysis of the study.
doi:10.1371/journal.pone.0101449.g001

function was investigated, with particular regards to adequate selection of patients.

Patients and Methods

Patients

Between May 2007 and December 2010, 42 patients with severe ischemic cardiomyopathy, presenting a decreased left ventricular ejection fraction (LVEF) >15% and <35%, were enrolled in our prospective phase I multicenter trial. The study protocol for all investigational sites (Department of Cardiovascular Surgery, University Duesseldorf; Clinic for Cardiothoracic, Transplantation and Vascular Surgery, Hannover Medical School; Department of Cardiac and Thoracic Vascular Surgery, University Hospital of Schleswig-Holstein, Luebeck; Department of Cardiac Surgery, University Heidelberg; each in Germany) was written according to the Declaration of Helsinki and approved by the national competent authority, the Paul Ehrlich Institute (reference number 188/01), as well as by the involved ethics committees. All patients gave written and informed consent to their participation in the trial. The inclusion and exclusion criteria for the study cohort are displayed in [Table 1]. A CONSORT (Consolidated Standards of Reporting Trials) flow diagram presenting the enrollment, intervention allocation, and data analysis is shown in [Figure 1]. Three out of 42 enrolled patients had to be excluded from the study due to the intraoperative necessity of technical deviations from the revascularization strategy, due to switching to an emergency operation, or due to missing data, respectively. [Table 2] presents the preoperative characteristics of the 39 included patients.

The EudraCT number of this study is 2005-004051-35 and the Current Controlled Trials number is ISRCTN49998633.

Methods

The protocol for this trial and supporting TREND checklist are available as Protocol S1 and Checklist S1.

Surgical procedure

A novel cell preparation protocol was used allowing for CABG with simultaneous separation and laser-supported transplantation of CD133+ cells derived from autologous bone marrow [9].

After inhalative anesthetization, patients underwent puncture of the iliac crest to aspirate an average volume of 281.9±7.4 ml bone marrow. Afterwards, the left internal thoracic artery and a great saphenous vein were prepared. The installation of extracorporeal circulation was followed by CABG with intermittent application of Calafiore blood cardioplegia.

Simultaneously, by using a CliniMACS cell separation system with a closed circuit (Miltenyi Biotech Inc., Bergisch Gladbach, Germany), CD133+ cells were isolated from the collected bone marrow. Due to considerations in terms of short handling time in parallel to the operation and highest possible cell vitality, the cell isolation was conducted in immediate vicinity of the operation theater under a separate clean laminar flow bench, which was approved by the Paul Ehrlich Institute. Quality analyses of the cell product were conducted by fluorescence-activated cell sorting measurements.

Following coronary artery reperfusion, the areas of hibernating myocardium technically not amenable to bypass grafting, which had been preoperatively selected by MRI and coronary angiography, were treated by creating an average number of 20.4±0.8 transmyocardial laser channels (CO_2 Heart Laser, PLC Medical Systems Inc., Milford, USA). The separated autologous CD133+ BMCs ($9.7\pm1.2 \times10^6$) were injected into these pretreated myocardial regions by the operating surgeon. Finally, the heart was weaned from cardiopulmonary bypass and the patient was

Table 2. Preoperative patients' characteristics.

**Age (y)**	66±1.4
**Male (%)**	84.6
**BMI (kg m^{-2})**	29±0.8
**Arterial hypertension (%)**	89.7
**Diabetes mellitus type II (%)**	51.3
**PAVD (%)**	23.1
**COPD (%)**	10.3
**Renal failure (%)**	17.9
**Previous myocardial infarction (%)**	56.4
**Previous coronary intervention (%)**	25.6
**Redo operation (%)**	10.3

BMI = body mass index; COPD = chronic obstructive pulmonary disease; PAVD = peripheral arterial vessel disease.
doi:10.1371/journal.pone.0101449.t002

transferred to the intensive care unit. The mean cut-suture time was 296.3±49.8 min.

An overview of the simultaneous procedures of surgery and cell preparation is displayed in [Figure 2].

Study endpoints

The primary composite endpoint of the present prospective trial, assessed at 3 months after surgical treatment, was the safety of the cell transplantation procedure, defined as the occurrence of major adverse cardiac or cerebrovascular events (MACCE: cardiac-related death, myocardial infarction, urgent or elective percutaneous coronary intervention, stroke, or transitory ischemic attack) or one of the following adverse events: secondary mitral valve damage or hospitalization for any adverse event thought to be related to cardiac function by the investigator.

The secondary endpoints comprised the above mentioned composite endpoint at 6 and 12 months as well as the following endpoints after 3, 6 and 12 months: adverse cardiac events, severity of angina as assessed by reduction of classes in the CCS grading scale, MRI-evaluated regional cardiac function and quality of life in comparison to baseline values.

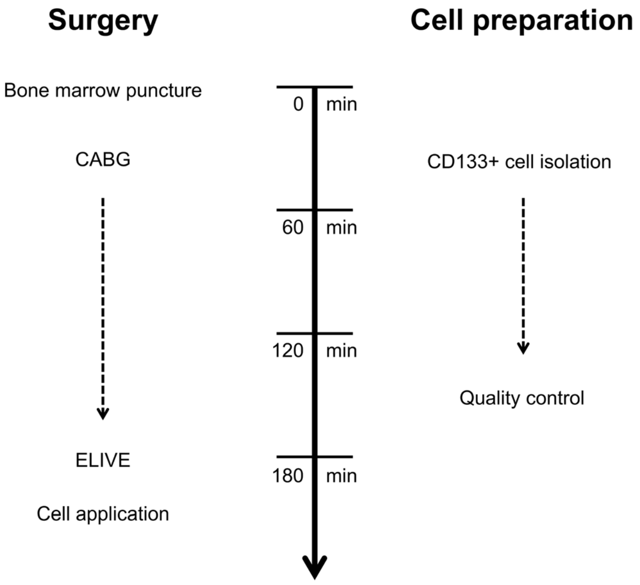

Figure 2. Surgical timeline of simultaneous CD133+ cell isolation and CABG with subsequent laser treatment(ELIVE)-supported cell transplantation. ELIVE, endogenous laser-induced ventricular enhancement.
doi:10.1371/journal.pone.0101449.g002

Perioperative examinations and follow-up

Cardiac MRI was used to evaluate segmental myocardial contractility as well as perfusion and to identify the areas of scar tissue preoperatively and 6 months after surgery. Data were pseudo-anonymized and analyzed in a blinded manner on a 1.5 Tesla MRI scanner (Avanto, Siemens, Forchheim, Germany) using a 17 myocardial segments system. While assessment of the global ejection fraction was part of the prospective trial, the segmental analyses were conducted separately. Qualitative regional cardiac function analyses were conducted on steady-state free procession cine images acquired in short axis views. After administration of 0.2 ml/kg body weight gadolinium–based contrast agent, perfusion imaging was performed during the first pass using a T1-weighted fast gradient echo sequence in three short axis imaging planes representing the basal, midventricular and apical myocardial segments. After administration of additional 0.2 ml/kg contrast material, delayed enhancement images were acquired after a delay of 10 minutes using an inversion-recovery gradient echo sequence. In the preoperative sequences, the amount of transmural delayed contrast agent enhancement as well as the percentaged number of segments with hibernating myocardium (hypokinetic or akinetic areas with perfusion deficit and lack of late enhancement) were determined. In case of contraindications against MRI (n = 3), cardiac computed tomography was conducted on a 64-row multidetector-CT (Somatom Sensation Cardiac, Siemens Healthcare, Forchheim, Germany). After an initial scout image and a timing bolus scan at the level of the aortic root with 20 ml of iodinated contrast agent (iomeprol, 400 g/cm^3, imeron 400, Bracco Imaging, Konstanz, Germany), an 80 ml bolus of contrast agent was power-injected at 5 ml/s followed by a 40 ml bolus of saline chaser. A retrospective ECG-gated volume dataset was acquired with 64 mm × 0.6 mm collimation, a gantry rotation time of 330 ms, a pitch of 0.2, tube voltage of 100–120 kV (weight-based nomogram), and a tube current of 312–370 mAs/rotation (scout-based automatic reference tube current selection–CareDose 4D, Siemens, Germany). To measure global functional parameters, axial 1.5 mm multiphase images were analyzed using commercially available software (Syngo Argus, Siemens, Germany).

Table 3. Endpoint events.

Primary endpoint	Secondary endpoint
I) Multi organ failure (48 hours)	
II) Septic shock (72 days)	
	III) Sudden cardiac death (6 months)

doi:10.1371/journal.pone.0101449.t003

Canadian Cardiovascular Society (CCS) grading was performed preoperatively as well as after 3, 6 and 12 months. Physical examinations, blood serum level analyses and electrocardiography were conducted preoperatively, after 12, 24 and 48 hours and at all follow-up examinations (3, 6 and 12 months). All adverse events were documented during the whole follow-up, were classified as MACCE if indicated, and their relationship to the cell therapy procedure was evaluated. In order to assess the quality of life, the Seattle Angina Questionnaire (SAQ) was used.

Statistics

Statistical analysis was performed with R version 2.15.1, and with SPSS version 20.0.0 (IBM, New York, USA). Descriptive statistics were calculated and are presented as mean values ± standard error of the mean for all continuous variables.

For direct comparisons of groups with continuous variables, paired or unpaired t-tests with or without Welch's correction were applied, as indicated. Regarding categorial variables, pairwise Fisher's exact tests were conducted. The blood serum levels at different time points were compared with each other by means of ANOVA with Tukey's posthoc test. The impact of preoperatively extensive transmural delayed enhancement and hibernating myocardium on the postoperative increase of LVEF was examined by a linear regression analysis. For all analytical statistics, p values <0.05 were assumed to indicate significance.

Results

Safety and feasibility

CD133+ cell isolation. The intraoperative isolation of CD133+ BMCs was successfully conducted in all patients. Flow cytometry analyses revealed the average number of transplanted cells to be $9.7 \pm 1.2 \times 10^6$, exhibiting a vitality of 93.2±0.9% and a purity of 82.6±2.3%.

Clinical outcome. Two patients suffered from severe adverse events which had to be classified as primary endpoint events: One patient developed a multi organ failure and died within the first 48 postoperative hours due to a hyperkalemia-related electromechanical dissociation, and another one had a severe septic shock with metabolic disorder and final ventricular fibrillation after 72 days. None of these adverse events was related to the cell therapy procedure. In another patient, which was an early drop-out due to emergency reconstruction of the mitral valve during the initial operation, a serious adverse event with possible relationship was observed and classified as serious adverse reaction by the coordinating investigator during the quality assurance procedure. In this patient, a sudden cardiac death occurred after drop-out, which can be seen as a typical consequence of the underlying disease.

Only one patient had a secondary endpoint event when dying of presumably sudden cardiac death within the first 6 months. Thorough assessment revealed no relationship to the cell therapy. After 12 months, no additional endpoint event had occurred, resulting in a total of three patients with endpoint events at month 12 [Table 3].

The CCS grading scale analysis revealed a significant decrease of the severity of angina after the surgical procedure. After 3, 6 and 12 months, the percentage of patients with CCS grades ≤II was 100% versus 35.1% preoperatively ($p<0.0001$ at each time point) [Table 4]. Analyzing the physical limitation scale of the SAQ, we found a significant increase of quality of life during the follow-up (40.2±1.4 after 3 months, 40.9±1.5 after 6 months, 43.7±1.0 after 12 months; each with $p<0.0001$ versus 28.4±1.5 preoperatively) [Figure 3].

Electrocardiography did not reveal any postoperatively new q-wave myocardial infarction or ST segment elevation. The blood serum level analyses during the follow-up revealed results as expected after cardiac surgery in patients with ischemic cardiomyopathy [Table 5].

Myocardial function

Left ventricular contractility. The LVEF significantly improved after surgery (29.7±1.9% versus 24.6±1.5% with $p = 0.004$ at 6 months). Moreover, the left ventricular enddiastolic volume was decreased (264.2±16.9 ml versus 284.4±14.8 ml with $p = 0.01$). After 6 months, the total number of hypokinetic segments was reduced (5.9±0.9 versus 6.6±0.9 preoperatively, $p = 0.296$), as well as the number of akinetic segments (3.2±0.5 versus 4.9±0.7 preoperatively, $p = 0.014$).

Prediction of the benefit of the CD133+ cell therapy. We aimed at examining whether patients with extensive myocardial scars profited less from the therapy. Interestingly, the preoperative global LVEF value was not correlated with the postoperative increase of the LVEF (slope 0.3±0.4 and $r^2 = 0.02$ with $p = 0.540$) [Figure 4A]. Therefore, we conducted more sophisticated regional analyses of myocardial function and viability based on a 17 myocardial segments model. In that context, we determined the correlation of the preoperative amount of transmural delayed contrast agent enhancement with the increase of the LVEF after 6 months. A linear regression analysis revealed a negative correlation of the two parameters (slope −1.8±0.7 and $r^2 = 0.25$ with $p = 0.024$) [Figure 4B]. Moreover, transmural delayed enhancement in more than three myocardial segments resulted in significantly lower increase of the LVEF after 6 months, as compared to patients with three or less affected segments (1.0±1.4% versus 10.0±4.3% with $p = 0.042$) [Figure 5]. A larger percentaged area of hibernating myocardium in the preoperative MRI was accompanied by a higher increase of the LVEF after 6 months. However, this correlation was not found to be significant (slope 0.2±0.2 and $r^2 = 0.1$ with $p = 0.237$) [Figure 4C]. Patients with more than 15% hibernating myocardium showed a larger postoperative increase of the LVEF than patients with less than 15% hibernating myocardium (9.2±4.8% versus 2.0±2.0% with $p = 0.063$).

Table 4. CCS grading before and after cell therapy.

CCS grading	Pre (n)	3 months * (n)	6 months * (n)	12 months * (n)
≤II	13	33	27	25
>II	24	0	0	0

At all postoperative time points, 100% of the patients showed CCS grades ≤II (versus 35.1% preoperatively). Pre, preoperatively; * $p<0.0001$ versus pre.
doi:10.1371/journal.pone.0101449.t004

Discussion

Safety and feasibility

The primary aim of the present multicenter trial was the examination of a combined therapeutic approach for patients with end-stage ischemic heart failure. This is the first clinical study of CABG with simultaneous, laser treatment(ELIVE)-supported CD133+ cell transplantation, and the results proved our protocol to be feasible and safe. Neither any CD133+ cell processing failure, nor any serious adverse events which had to be assessed as at least probable procedure-related events were observed. Two serious adverse events with possible procedure-related relationship were reported in the trial, however, in one patient re-assessment after the follow-up revealed no relationship, and in the other patient, a sudden cardiac death occurred after drop-out, which can be seen as a typical consequence of the underlying disease. While in most previous BMC transplantation studies, the bone marrow was harvested pre-interventionally, the approach of the present study features intraoperative cell isolation and processing without the necessity of additional anesthetization, cultivation or storage of the cell product, significantly simplifying processing logistics [10]. Albeit using a time-optimized accelerated procedure protocol, the procedure resulted in a high-quality cell product in terms of purity as well as vitality. This approach was presented for the first time in a small cohort study in patients with intractable ischemic heart disease who received laser treatment and CD133+ cell transplantation [11].

The rationale of conducting ELIVE before cell transplantation was to create an environment by which survival, growth and potential function-restoring effects of the injected cells may be supported, especially by inducing angiogenesis. In porcine models of chronic myocardial ischemia as well as acute myocardial infarction, laser treatment was previously shown to improve local angiogenesis as well as the survival of administered mesenchymal stem cells into the infarcted region [12–15].

Clinical outcome

As a result of the combined surgical procedure, the CCS grades were significantly reduced and the quality of life was improved 3, 6 and 12 months after surgery. Both effects may partially be attributed to coronary revascularization, which was performed in all these patients. However, all study patients suffered from severe heart failure with ischemic areas of viable myocardium which were technically ineligible for CABG. Nevertheless, we found a significant decrease of clinical ischemia symptoms with no residual angina after 6 months. While many studies have shown short-term improvement of symptoms after laser treatment in patients with refractory angina, controversial data have been reported on the

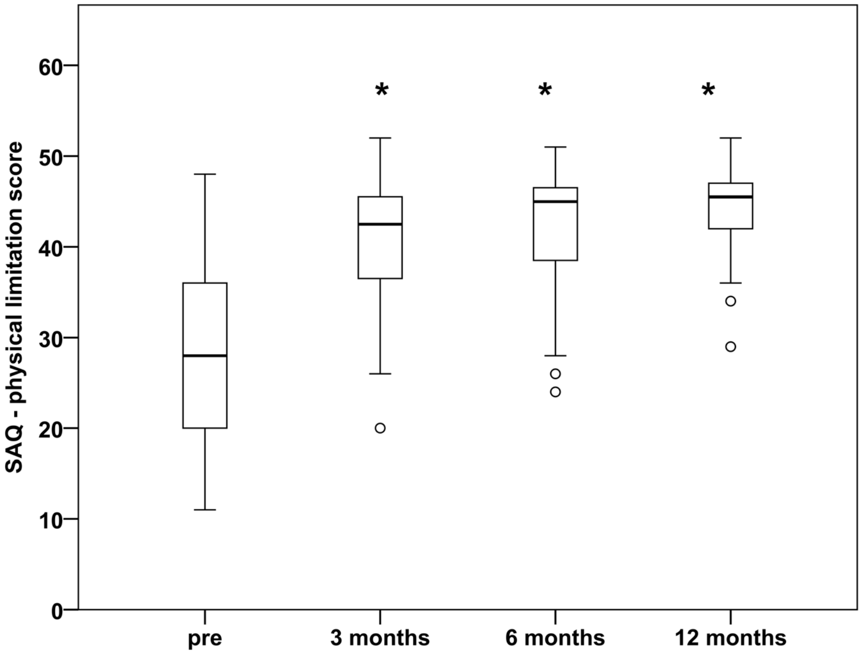

Figure 3. Quality of life after cell therapy. At all postoperative time points, the physical limitation score of the Seattle Angina Questionnaire (SAQ) was significantly improved (40.2±1.4 after 3 months, 40.9±1.5 after 6 months, 43.7±1.0 after 12 months, versus 28.4±1.5 preoperatively). Pre, preoperatively. Separated values, outliers included in the statistical analysis. Asterisk, $p<0.0001$ versus pre.
doi:10.1371/journal.pone.0101449.g003

Table 5. Blood serum level analyses.

Parameter	Pre	12 h	24 h	48 h	3 mo	6 mo	12 mo
**Hemoglobin (g/dl)**	13.9±0.2	11.0±0.3 ***	10.9±0.3 ***	10.8±0.3 ***	13.5±0.2	14.1±0.4	14.2±0.3
**Hematocrit (%)**	42.1±0.7	34.0±1.1 ***	33.3±0.8 ***	32.5±0.8 ***	40.9±0.7	41.6±0.9	42.8±0.9
**Potassium (mmol/l)**	4.5±0.1	4.8±0.1	4.8±0.1	4.7±0.1	4.6±0.1	4.6±0.1	4.7±0.1
**LDH (U/l)**	203.6±7.5	337.2±28.7 **	355.1±53.6 **	265.8±18.1	191.4±6.9	178.1±7.3	201.5±10.9
**CKMB (U/l)**	11.7±1.0	33.8±4.5 **	35.2±5.2 **	24.4±4.8	11.9±1.0	9.8±1.1	13.8±1.8
**Troponin T (ng/ml)**	0.1±0.1	1.0±0.2 *	1.2±0.3 **	0.6±0.2	0.01±0.001	0.01±0.001	0.03±0.01
**Creatinine (mg/dl)**	1.2±0.1	1.2±0.1	1.4±0.1	1.4±0.1	1.3±0.1	1.1±0.1	1.2±0.1
**CRP (mg/dl)**	3.0±1.1	9.3±1.2	17.5±1.5 **	26.9±6.3 ***	2.4±1.1	0.9±0.3	0.5±0.1

All postoperative values were statistically compared with the referring baseline value. LDH, lactate dehydrogenase; CKMB, creatine kinase-myocardial band; pre, preoperatively; *p<0.05; **p<0.01; ***p<0.001.
doi:10.1371/journal.pone.0101449.t005

long-term outcome including studies not confirming clinical or functional benefit beyond 18 month after TMLR only [16,17]. These findings underline single laser treatment to be an end-stage option for patients with severe heart failure. For this reason, combined approaches utilizing TMLR and cell therapy have been proposed aiming at the cellular component to beneficially affect the diseased myocardium in the long-term, predominantly by paracrine effects on resident cardiac cells. Among other cell lines, CD133+ and CD34+ cells were reported to reduce the CCS grades in patients with ischemic cardiomyopathy [2,18]. Combined laser and BMC therapy also exerted positive effects on refractory angina [19].

In our study, no patient died due to cardiac reasons, although all patients were initially suffering from end-stage ischemic cardiomyopathy. BMC treatment in patients with chronic ischemia was previously reported to reduce the mortality even 5 years after intracoronary cell delivery [20]. For patients with acute myocardial infarction, the BOOST trial could not confirm the superiority of intracoronary BMC transplantation in terms of mortality after 5 years [21]. However, large trials with long follow-up periods are necessary in order to further evaluate the potential of cell therapy to improve the survival of patients with chronic ischemia.

Improvement of myocardial function

The combined approach of ELIVE and CD133+ cell therapy in CABG patients of the present trial beneficially affected the left ventricular function in severe ischemic cardiomyopathy. In a previous study, Stamm et al. described a significant postoperative improvement of the LVEF 6 months after CD133+ cell transplantation in CABG patients with moderately reduced LVEF [10]. However, the same group reported that with regard to the LVEF, combined CABG and cell therapy were not superior to single CABG beyond 6 months after surgery [22]. In fact, they found a LVEF <40% and a time period of 7 to 12 weeks between myocardial infarction and surgery to be predictors of an improvement of the LVEF of at least 5%. Similarly, intracoronary infusion of CD133+ cells in patients with chronic myocardial infarction was reported to improve the left ventricular function [4]. In opposite to these studies on CD133+ cells, the transplantation of unfractionated BMCs in chronically ischemic myocardium failed to show functional improvement in several studies [3]. One year after having injected mononuclear BMCs in the infarction border zone in CABG patients with a LVEF ≤45%, the investigators could not find a beneficial effect of cell therapy on the LVEF or the wall thickening measured by MRI, when compared to the control group with CABG only [23]. However, when analyzing the myocardial scar size in the cell administration areas, a significant decrease was observed in the BMC group. A study comparing intramyocardial as well as intracoronary BMC application in CABG patients with CABG only did not reveal any significant improvement in several parameters of global and regional cardiac function after 6 months, whereas intramyocardial injection showed a remarkable trend towards reduced scar size as well as improvement of the LVEF, when compared with intracoronary injection or controls [24].

Trials examining intracoronary transplantation of BMCs in patients after acute myocardial infarction and successful interventional revascularization resulted in controversial functional results: Two years after intracoronary cell administration, the baseline-adjusted LVEF in the BMC group of the AMI-REPAIR trial was reported to be significantly improved versus control patients [25]. After an initial increase of the LVEF in the BMC group of the BOOST trial (6 months), Meyer et al. observed no significant

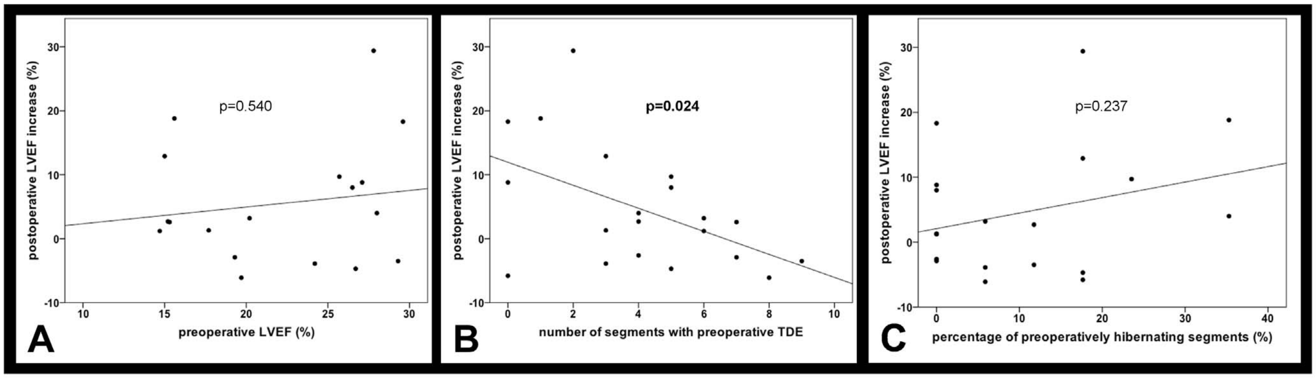

Figure 4. Preoperative indicators for the improvement of the LVEF. While the preoperative global LVEF values were not correlated with the postoperative increase of the LVEF (A), we found an inverse correlation of the preoperative amount of transmural delayed enhancement (TDE) with the postoperative increase of the LVEF (B). The association of a preoperatively larger percentaged area of hibernating myocardium with a higher increase of the LVEF was not statistically significant (C).
doi:10.1371/journal.pone.0101449.g004

functional improvement by intracoronary cell therapy after 18 and 61 months, whereas the baseline LVEF values were higher than in the AMI-REPAIR trial [21]. The SWISS-AMI trial aimed at determining the optimal time point of BMC administration after acute myocardial infarction. However, neither by early (5–7 days) nor by late (3–4 weeks) intracoronary cell therapy, an improvement of the LVEF could be achieved after 4 months [26]. Intracoronary cell application in the BONAMI trial resulted in enhanced myocardial viability after 3 months, measured by single-photon-emission computed tomography [27]. However, the LVEF was not increased as compared to control patients. A very similar study design was applied to the HEBE trial and the TIME trial, which likewise did not result in an improvement of the LVEF after 4 and 6 months, respectively [28,29]. As opposed to the implications of the BONAMI trial, no post-interventional reduction of dyskinetic myocardial segments was observed. Patients in the cell therapy and the control group of the BONAMI trial had a mean baseline LVEF of 35.6% and 37.0%, respectively, while in the HEBE trial, the values accounted for 43.7% and 41.7% in the therapy groups and 42.4% in the control group.

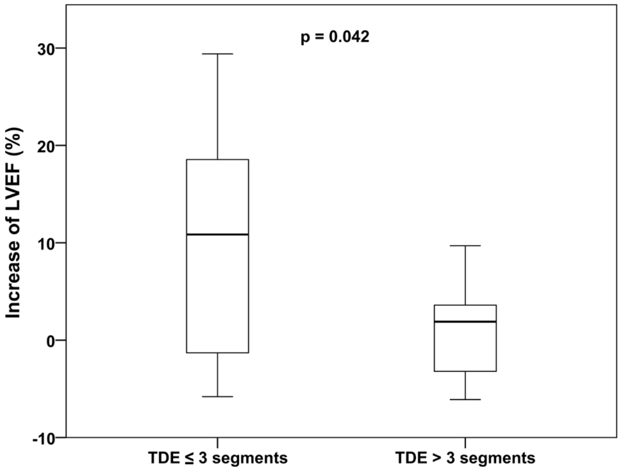

Figure 5. Transmural delayed enhancement (TDE) and the postoperative increase of the LVEF. TDE in more than three myocardial segments (at screening MRI) resulted in significantly less increase of the LVEF after 6 months, as compared to patients with three or less affected segments (1.0 ± 1.4 versus 10.0 ± 4.3, $p=0.042$).
doi:10.1371/journal.pone.0101449.g005

In a previous publication on the technical approach of combined CABG, laser treatment and CD133+ cell therapy, we have reported exemplary LVEF data which had been measured by 2D echocardiography in patients having been included in the study at early time points [9]. This subset of patients even showed a LVEF increase from 25% preoperatively to 40% after 6 months. Analysis of the echocardiography-based LVEF of all study patients at the end of the trial resulted in an increase from $27.7\pm0.9\%$ preoperatively to $37.6\pm1.4\%$ after 6 months ($p<0.001$). Although the final study evaluation based on MRI could not confirm this extent of functional improvement, still a significant increase in LVEF was observed (30% after 6 months vs. 25% preoperatively). Besides the fact that the previous publication had included only some of the study patients which had already been followed-up at that time point, the evaluation method may explain the different results. While 2D echocardiography data can be observer-dependent to a large extent, MRI is regarded to be the gold standard for global as well as regional functional evaluation of ischemic myocardium [30–32].

Adequate selection of patients undergoing cell therapy

The controversial clinical reports on functional improvement by cell therapy of ischemic myocardium raise the question of adequate selection of patients undergoing myocardial cell therapy. In particular, preoperative parameters which are correlated to the post-therapeutic improvement of myocardial function in ischemic cardiomyopathy are required.

With regard to patients' selection, Yerebakan et al. had reported that patients with a LVEF <40% profit more from CD133+ cell transplantation [22]. In the present study cohort of patients with LVEF values <35%, the preoperative LVEF did not influence the functional benefit of the cell therapy. Especially in terms of severe chronic ischemia with large myocardial scar tissue areas, more sophisticated examinations seem to be necessary to select the right patients for cell therapy.

Moreover, adequate sites of injection of the cell suspension have to be chosen. In order to address both aspects, we determined all myocardial segments with transmural delayed contrast agent enhancement in the preoperative MRI measurements, indicating myocardial scar tissue [33]. These analyses revealed that patients with less increase of the LVEF after surgical cell therapy had larger

areas of transmural delayed enhancement in the preoperative MRI. A total number of more than three out of 17 segments with transmural delayed enhancement was shown to be significantly associated with a less beneficial functional outcome. Therefore, the extent of transmural delayed enhancement may be used as a preoperative predictor of the effectiveness of cell therapy in chronically ischemic myocardium.

Limitations of the study

The present prospective study was primarily designed as a safety and feasibility trial without control group. Thus, our combined approach of CABG, ELIVE and cell therapy does not allow for separately discriminating the actual effects of ELIVE and cell therapy. However, laser treatment is strongly assumed to establish a supportive environment for cell therapy, while the transplanted cells may induce restoration of the myocardial function. In order to track the *in vivo* fate of the administered cells and to correlate this information with observed clinical benefits, modern imaging techniques based on MRI or positron emission tomography should be additionally considered in future trials [34].

Conclusions

In the present multicenter trial, we established a safe protocol of intraoperative CD133+ cell isolation and laser-supported transplantation in CABG patients with end-stage ischemic cardiomyopathy. This approach resulted in relief from angina and significant improvement of quality of life as well as of the myocardial function measured by left ventricular ejection fraction and enddiastolic dilation. However, the beneficial results obtained from this pilot study require further evaluation in a randomized efficacy trial. More interestingly, we elucidated the preoperative amount of transmural delayed enhancement to be inversely correlated with the postoperative increase of the left ventricular function. Thereby, MRI-guided selection of patients may be a worthwhile tool to improve the effectiveness of laser-supported myocardial cell therapy. In this context, large-scale studies in patients with moderately as well as severely impaired myocardial function are necessary to confirm adequate selection criteria for patients undergoing cell therapy.

Supporting Information

Protocol S1 Clinical trial protocol of this study.
(PDF)

Checklist S1 TREND statement checklist.
(PDF)

Acknowledgments

The authors gratefully thank Dr. med. Johannes Fischer (Institute for Transplantation Diagnostics and Cell Therapeutics, Heinrich Heine University, Medical Faculty, Duesseldorf, Germany) for his contribution regarding the flow cytometry analyses.

Author Contributions

Conceived and designed the experiments: AA MH CO HMK. Performed the experiments: AA MH PK LP AM AK IK HHS HMK. Analyzed the data: AA MH PK LP DH CO MYE HMK. Contributed reagents/materials/analysis tools: CO IK HHS HMK. Wrote the paper: AA MH CO MYE HMK.

References

1. Sanganalmath SK, Bolli R (2013) Cell therapy for heart failure: a comprehensive overview of experimental and clinical studies, current challenges, and future directions. Circ Res 113: 810–834.
2. Klein HM, Ghodsizad A, Marktanner R, Poll L, Voelkel T, et al. (2007) Intramyocardial implantation of CD133+ stem cells improved cardiac function without bypass surgery. Heart Surg Forum 10: E66–E69.
3. Menasché P (2008) Cardiac cell therapy trials: chronic myocardial infarction and congestive heart failure. J Cardiovasc Transl Res 1: 201–206.
4. Manginas A, Goussetis E, Koutelou M, Karatasakis G, Peristeri I, et al. (2007) Pilot study to evaluate the safety and feasibility of intracoronary CD133(+) and CD133(−) CD34(+) cell therapy in patients with nonviable anterior myocardial infarction. Catheter Cardiovasc Interv 69: 773–781.
5. Strauer BE, Steinhoff G (2011) 10 years of intracoronary and intramyocardial bone marrow stem cell therapy of the heart: from the methodological origin to clinical practice. J Am Coll Cardiol 58: 1095–1104.
6. Jeevanantham V, Butler M, Saad A, Abdel-Latif A, Zuba-Surma EK, et al. (2012) Adult bone marrow cell therapy improves survival and induces long-term improvement in cardiac parameters: a systematic review and meta-analysis. Circulation 126: 551–568.
7. Li W, Tanaka K, Chiba Y, Kimura T, Morioka K, et al. (2003) Role of MMPs and plasminogen activators in angiogenesis after transmyocardial laser revascularization in dogs. Am J Physiol Heart Circ Physiol 284: H23–H30.
8. Klein HM, Ghodsizad A, Borowski A, Saleh A, Draganov J, et al. (2004) Autologous bone marrow-derived stem cell therapy in combination with TMLR. A novel therapeutic option for endstage coronary heart disease: report on 2 cases. Heart Surg Forum 7: E416–E419.
9. Klein HM, Assmann A, Lichtenberg A, Heke M (2010) Intraoperative CD133+ cell transplantation during coronary artery bypass grafting in ischemic cardiomyopathy. MMCTS (0809):3947.
10. Stamm C, Kleine HD, Choi YH, Dunkelmann S, Lauffs JA, et al. (2007) Intramyocardial delivery of CD133+ bone marrow cells and coronary artery bypass grafting for chronic ischemic heart disease: safety and efficacy studies. J Thorac Cardiovasc Surg 133: 717–725.
11. Babin-Ebell J, Sievers HH, Charitos EI, Klein HM, Jung F, et al. (2010) Transmyocardial laser revascularization combined with intramyocardial endothelial progenitor cell transplantation in patients with intractable ischemic heart disease ineligible for conventional revascularization: preliminary results in a highly selected small patient cohort. Thorac Cardiovasc Surg 58: 11–16.
12. Hughes GC, Kypson AP, Annex BH, Yin B, St Louis JD, et al. (2000) Induction of angiogenesis after TMR: a comparison of holmium: YAG, CO2, and excimer lasers. Ann Thorac Surg 70: 504–509.
13. Hughes GC, Biswas SS, Yin B, Baklanov DV, Annex BH, et al. (2002) A comparison of mechanical and laser transmyocardial revascularization for induction of angiogenesis and arteriogenesis in chronically ischemic myocardium. J Am Coll Cardiol 39: 1220–1228.
14. Fuchs S, Baffour R, Vodovotz Y, Shou M, Stabile E, et al. (2002) Laser myocardial revascularization modulates expression of angiogenic, neuronal, and inflammatory cytokines in a porcine model of chronic myocardial ischemia. J Card Surg 17: 413–424.
15. Patel AN, Spadaccio C, Kuzman M, Park E, Fischer DW, et al. (2007) Improved cell survival in infarcted myocardium using a novel combination transmyocardial laser and cell delivery system. Cell Transplant 16: 899–905.
16. Schneider J, Diegeler A, Krakor R, Walther T, Kluge R, et al. (2001) Transmyocardial laser revascularization with the holmium:YAG laser: loss of symptomatic improvement after 2 years. Eur J Cardiothorac Surg 19: 164–169.
17. Horvath KA (2008) Transmyocardial laser revascularization. J Card Surg 23: 266–276.
18. Losordo DW, Henry TD, Davidson C, Sup Lee J, Costa MA, et al. (2011) Intramyocardial, autologous CD34+ cell therapy for refractory angina. Circ Res 109: 428–436.
19. Reyes G, Allen KB, Aguado B, Duarte J (2009) Bone marrow laser revascularisation for treating refractory angina due to diffuse coronary heart disease. Eur J Cardiothorac Surg 36: 192–194.
20. Strauer BE, Yousef M, Schannwell CM (2010) The acute and long-term effects of intracoronary Stem cell Transplantation in 191 patients with chronic heARt failure: the STAR-heart study. Eur J Heart Fail 12: 721–729
21. Meyer GP, Wollert KC, Lotz J, Pirr J, Rager U, et al. (2009) Intracoronary bone marrow cell transfer after myocardial infarction: 5-year follow-up from the randomized-controlled BOOST trial. Eur Heart J 30: 2978–2984.
22. Yerebakan C, Kaminski A, Westphal B, Donndorf P, Glass A, et al. (2011) Impact of preoperative left ventricular function and time from infarction on the long-term benefits after intramyocardial CD133(+) bone marrow stem cell transplant. J Thorac Cardiovasc Surg 142: 1530–1539.
23. Pätilä T, Lehtinen M, Vento A, Schildt J, Sinisalo J, et al. (2014) Autologous bone marrow mononuclear cell transplantation in ischemic heart failure: A prospective, controlled, randomized, double-blind study of cell transplantation combined with coronary bypass. J Heart Lung Transplant pii:S1053–2498(14)00971-1. doi: 10.1016/j.healun.2014.02.009 [Epub ahead of print].

24. Ang KL, Chin D, Leyva F, Foley P, Kubal C, et al. (2008) Randomized, controlled trial of intramuscular or intracoronary injection of autologous bone marrow cells into scarred myocardium during CABG versus CABG alone. Nat Clin Pract Cardiovasc Med 5: 663–670.
25. Assmus B, Rolf A, Erbs S, Elsässer A, Haberbosch W, et al. (2010) Clinical outcome 2 years after intracoronary administration of bone marrow-derived progenitor cells in acute myocardial infarction. Circ Heart Fail 3: 89–96.
26. Sürder D, Manka R, Lo Cicero V, Moccetti T, Rufibach K, et al. (2013) Intracoronary Injection of Bone Marrow Derived Mononuclear Cells, Early or Late after Acute Myocardial Infarction: Effects on Global Left Ventricular Function Four months results of the SWISS-AMI trial. Circulation 127: 1968–1979.
27. Roncalli J, Mouquet F, Piot C, Trochu JN, Le Corvoisier P, et al. (2011) Intracoronary autologous mononucleated bone marrow cell infusion for acute myocardial infarction: results of the randomized multicenter BONAMI trial. Eur Heart J 32: 1748–1757.
28. Hirsch A, Nijveldt R, van der Vleuten PA, Tijssen JG, van der Giessen WJ, et al. (2011) Intracoronary infusion of mononuclear cells from bone marrow or peripheral blood compared with standard therapy in patients after acute myocardial infarction treated by primary percutaneous coronary intervention: results of the randomized controlled HEBE trial. Eur Heart J 32: 1736–1747.
29. Traverse JH, Henry TD, Pepine CJ, Willerson JT, Zhao DX, et al. (2012) Effect of the use and timing of bone marrow mononuclear cell delivery on left ventricular function after acute myocardial infarction: the TIME randomized trial. JAMA 308: 2380–2389.
30. Grothues F, Smith GC, Moon JC, Bellenger NG, Collins P, et al. (2002) Comparison of interstudy reproducibility of cardiovascular magnetic resonance with two-dimensional echocardiography in normal subjects and in patients with heart failure or left ventricular hypertrophy. Am J Cardiol 90: 29–34.
31. Pennell DJ, Sechtem UP, Higgins CB, Manning WJ, Pohost GM, et al. (2004) Clinical indications for cardiovascular magnetic resonance (CMR): Consensus Panel report. J Cardiovasc Magn Reson 6: 727–765.
32. Klein C, Nekolla SG, Bengel FM, Momose M, Sammer A, et al. (2002) Assessment of myocardial viability with contrast-enhanced magnetic resonance imaging: comparison with positron emission tomography. Circulation 105: 162–167.
33. Vogel-Claussen J, Rochitte CE, Wu KC, Kamel IR, Foo TK, et al. (2006) Delayed enhancement MR imaging: utility in myocardial assessment. Radiographics 26: 795–810.
34. Nyolczas N1, Charwat S, Posa A, Hemetsberger R, Pavo N, et al. (2009) Tracking the migration of cardially delivered therapeutic stem cells in vivo: state of the art. Regen Med 4: 407–422.
